# Design Features to Accelerate the Higher-Order Assembly of DNA Origami on Membranes

**DOI:** 10.1021/acs.jpcb.1c07694

**Published:** 2021-11-24

**Authors:** Yusuf Qutbuddin, Jan-Hagen Krohn, Gereon A. Brüggenthies, Johannes Stein, Svetozar Gavrilovic, Florian Stehr, Petra Schwille

**Affiliations:** † Department of Cellular and Molecular Biophysics, Max Planck Institute of Biochemistry, Am Klopferspitz 18, D-82152 Martinsried, Germany; ‡ Exzellenzcluster ORIGINS, Boltzmannstrasse 2, D-85748 Garching, Germany

## Abstract

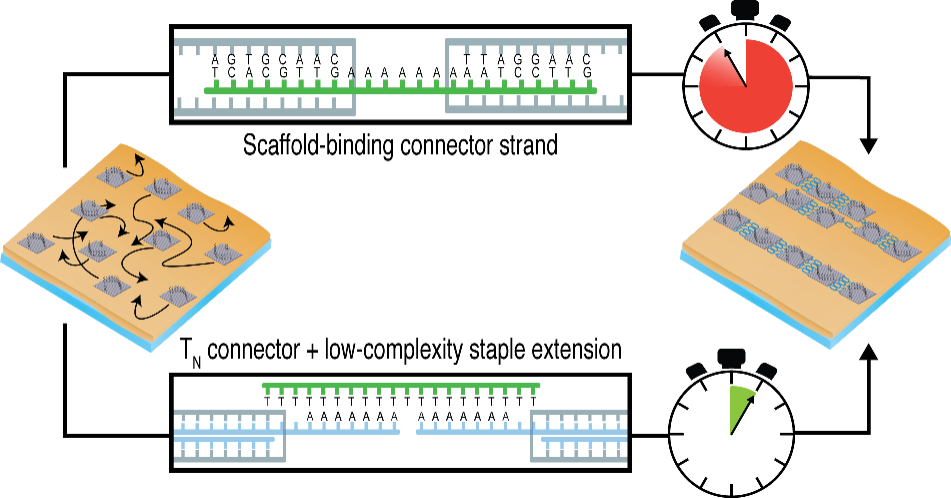

Nanotechnology often exploits DNA origami nanostructures assembled into even larger superstructures up to micrometer sizes with nanometer shape precision. However, large-scale assembly of such structures is very time-consuming. Here, we investigated the efficiency of superstructure assembly on surfaces using indirect cross-linking through low-complexity connector strands binding staple strand extensions, instead of connector strands binding to scaffold loops. Using single-molecule imaging techniques, including fluorescence microscopy and atomic force microscopy, we show that low sequence complexity connector strands allow formation of DNA origami superstructures on lipid membranes, with an order-of-magnitude enhancement in the assembly speed of superstructures. A number of effects, including suppression of DNA hairpin formation, high local effective binding site concentration, and multivalency are proposed to contribute to the acceleration. Thus, the use of low-complexity sequences for DNA origami higher-order assembly offers a very simple but efficient way of improving throughput in DNA origami design.

## Introduction

Over the past 15 years, the development of DNA origami technology led to huge advances in the field of structural DNA nanotechnology, as it allows straightforward construction of large and complex nanostructures.^1^ This is obtained by forcing long single-stranded DNA (ssDNA) “scaffold” strands into programmed conformations using many short “staple” strands. Diverse structures are possible, and multiple site-specific functionalizations can be introduced into a single structure with few-nanometer resolution.^2,3^ Applications include single-molecule observation of chemical reactions,^4^ positioning of nanoparticles for nanophotonics,^5^ design of sensitive and specific biosensors,^6^ and many others. Recent examples of DNA origami nanostructures designed in our lab include benchmark targets for single-molecule method development,^7^ curved nanostructures to deform membranes,^8^ or nanostructures serving as passive cargo to study transport processes in reaction–diffusion systems.^9^

The structural complexity allowed by the DNA origami technology is essentially limited by the length of the scaffold strand, typically 7–8 kb bacteriophage genomes. Even with cutting-edge strategies to increase the scaffold length up to 10 kb and modify it for different applications,^10,11^ it is still challenging to produce DNA origami in sizes above 100 nm with high yield. To arrive at larger structures, the very first publication of the DNA origami technology already introduced the idea of cross-linking origami “monomer” particles into higher-order structures.^1^ Nowadays, quite large and complex higher-order DNA origami structures (“superstructures”) are being used for nanometer-precise positioning of structures over micrometer scales,^12,13^ molecular “tubing” systems for linear transport of cargo,^14^ or the encapsulation of cargo that itself is tens of nanometers in diameter.^15^

There are multiple strategies for assembling DNA origami superstructures. The most common ones exploit direct DNA–DNA binding, either sticky-end hybridization^16^ or blunt-end stacking.^17^ We focus on sticky-end hybridization strategies in the present manuscript: First, as sticky-end hybridization exploits Watson–Crick base pairing, the association is specific and programmable.^12^ Second, sticky-end hybridization can be induced in a time-controlled manner by first preparing samples from DNA origami monomers and then cross-linking them by adding “connector strands”.^18^ Notably, programmability and time control are in principle also possible with blunt-end stacking but are more restricted.^17,19^ Sticky-end hybridization is typically performed by two alternative approaches: One option is to directly prepare one origami species with staple strands that are extended with sticky ends binding to sequences in another origami, either directly in the scaffold, or in staple extensions.^12,20^ Alternatively, to control the timing of association, one can prepare ssDNA stretches on the origami nanostructures and later add separate connector strands to bind and cross-link those ssDNA stretches *in situ*.^16,18^ Here we will address the latter strategy (Figure 2), as DNA superstructure assembly with time-controlled onset is valuable for synthetic biology applications, such as mimicking cytoskeleton assembly in order to probe the response of *in vitro* reconstituted proteins to changes in their environment. Time control is also accessible through photoactivation schemes,^21^ but this requires additional functionalization of oligomers. We aimed for a radically simple design for time-controlled DNA origami superstructure assembly, avoiding multistep assemblies,^12,14^ special buffer requirements,^19^ or non-DNA functionalizations.^21^

To allow time-controlled formation of DNA origami superstructures, the effective association rates after reaction initiation should be as high as possible. Past studies of DNA origami superstructures were often quite unsatisfactory in this regard, usually requiring incubation times in the order of 1 h or more,^22^ up to overnight incubation.^16,23^ Several ways to accelerate association have been identified. One option is multivalent binding between origami monomers to facilitate nucleation.^20,24^ Specifically, for origami in 2D systems, increasing DNA origami monomer diffusion coefficients by adding monovalent cations and/or depositing particles on a fluid lipid bilayer rather than on a solid support accelerates assembly.^18,19,23^ Additional acceleration comes from precisely matched and rigid geometries of the associating staple extensions to accelerate transition from monovalent binding nucleation to multivalent full binding.^20^ Importantly, at least in solution, association rates for DNA origami dimerization reach values comparable to typical association rates for free DNA oligonucleotides.^20^ This indicates that increasing effective association rates of the hybridization reaction itself may yield an additional gain in DNA origami superstructure assembly speed. With this idea in mind, we reasoned that recent developments toward increasing hybridization on-rates in DNA point accumulation for imaging in nanoscale topography (DNA-PAINT) microscopy could be transferred to accelerate DNA origami superstructure assembly.^25^

**Figure 1:**
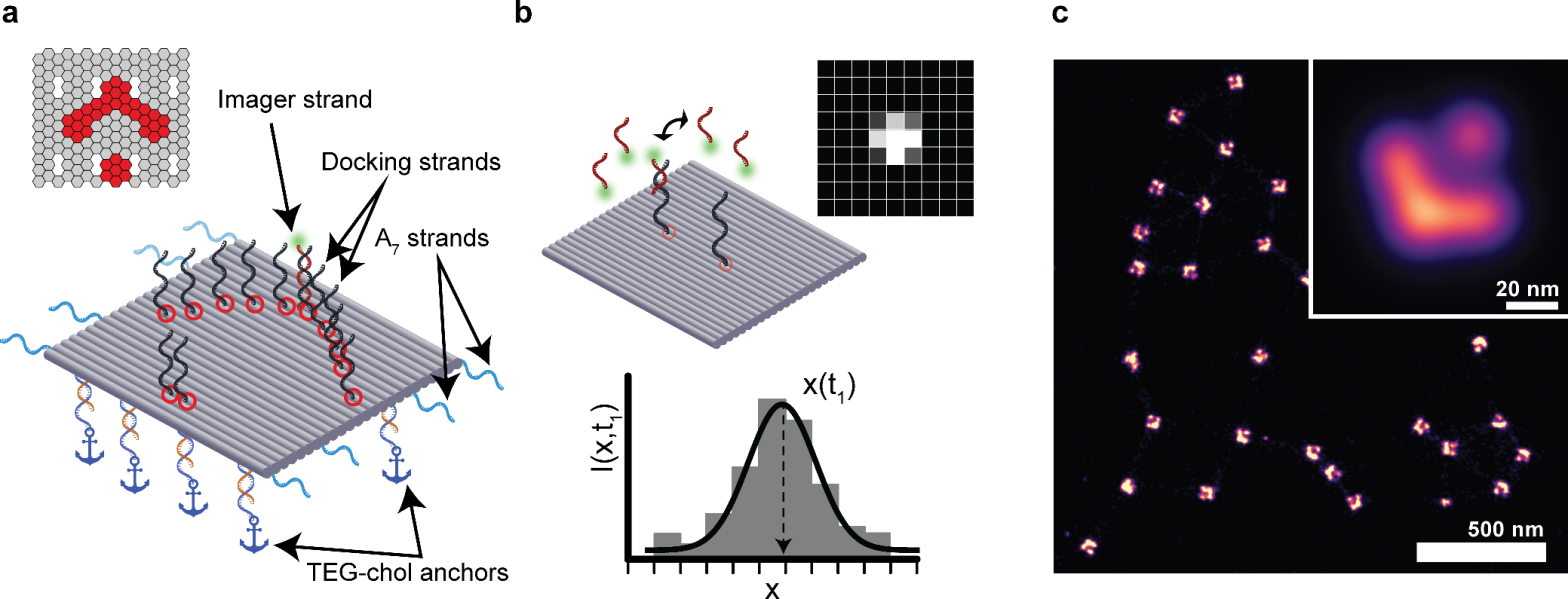
Design of DNA origami nanostructure used in this study. (a) Design schematic (elements not to scale). A 24-helix bundle is functionalized with a 36 docking sites for imager strands. Only a subset of these is shown for clarity, the Picasso Design^26^ schematic in the corner shows the true arrangement. Additionally, the particle is functionalized for membrane binding (orange extensions binding dark-blue “anchor” sequences) and lateral extensions for linear cross-linking (light-blue). (b) DNA-PAINT super-resolution imaging. Imager strands reversibly bind to the docking sites on the particle, successively highlighting them and allowing their super-resolved position determination. (c) Experimental DNA-PAINT data from surface-immobilized DNA origami particles, with arrows shapes clearly resolved on many particles. Inset shows an average image from 32 901 particles.

DNA-PAINT (Figure 1b) super-resolution microscopy is an implementation of single-molecule localization microscopy (SMLM) in which fluorophore-conjugated “imager strand” oligonucleotides reversibly bind to “docking sites” on the structure of interest. With low concentrations of imager strands, only a sparse random subset of docking sites is labeled at each time point, allowing their imaging in the single-molecule regime. Acquisition of thousands of frames and subsequent emitter point spread function fitting allows reconstruction of a super-resolved map of docking site coordinates.^26−28^ Recent improvements in DNA-PAINT acquisition speed focus on improved docking site design. Specifically, docking sites with low-complexity sequences, i.e., repeats of a short sequence motif such as [CTC]_*N*_, were found to be superior: These offer a large number of overlapping imager strand binding sites and thus increase the effective association rates for imager strand binding.^25^ The same strategy can also be used in single-particle tracking (SPT) of sparse sets of DNA origami particles.^28^ In this case, a long docking strand and a high concentration of imager strands yield unusually long tracks due to continuous replacement of bleached imager strands, circumventing photobleaching limitations to track duration.^29^

We thus set out to characterize two different sticky-end-based DNA origami superstructure assembly approaches in a lipid membrane-anchored 2D system. We use fluorescence techniques including single-particle tracking (SPT), DNA-PAINT, and image correlation analysis, complemented by atomic force microscopy (AFM), to characterize the assembly kinetics and the resulting structures. To this end, we employ a simple, stochastically assembling DNA origami superstructure based on rectangular monomers.^1,26^ We functionalized this DNA origami with staple extensions for cross-linking using low-complexity sequence connector strands to assemble superstructures *in situ* rather than preforming them in solution. We demonstrate assembly kinetics that are 1 order of magnitude faster than more traditional approaches by using low-complexity sequence connector strands. We discuss effects contributing to the acceleration, in particular the influence of length of the used sticky end. Our results provide useful insights for future experiments that require rapid cross-linking of DNA origami superstructures.

## Materials and Methods

Unless specified otherwise, chemicals were purchased from Sigma-Aldrich/Merck. DNA oligonucleotide sequences can be found in the Supporting Information.

### Buffer Compositions

DNA origami folding buffer: 12.5 mM MgCl_2_, 10 mM tris, 1 mM EDTA, pH 8.0. Buffer A: 100 mM NaCl, 10 mM tris, pH 8.0. Buffer B: 10 mM MgCl_2_, 5 mM tris, 1 mM EDTA, pH 8.0. Buffer D: 140 mM NaCl, 7.5 mM MgCl_2_, 20 mM tris, 0.75 mM EGTA, pH 7.6. SLB formation buffer: 150 mM KCl, 5 mM MgCl_2_, 25 mM tris, pH 7.5. SLB washing buffer: 150 mM KCl, 25 mM tris, pH 7.5. AFM imaging buffer: 40 mM MgCl_2_, 5 mM tris, pH 7.5.

### Origami Folding and Purification

DNA origami were designed using Picasso Design software,^26^ and modified using caDNAno.^30^ Scaffold DNA (p7249, tilibit nanosystems, 10 nM in folding buffer) was mixed with a 10-fold molar excess of unmodified staple strands or staple strands with extensions for tetraethyleneglycol–cholesterol (TEG-chol)-anchoring to membranes. Staple strands with DNA-PAINT docking site extensions, the adapter sequence for the “tracking handle”, or A_7_ cross-linking extensions were added in a 100-fold molar excess. The folding reaction was performed via melting for 5 min at 80 °C and temperature ramping from 60 to 4 °C over 3 h. The folded origami were PEG-purified by two cycles of dilution (1:1 in folding buffer containing additional 15% *w*/*v* PEG-8000 (89510) and 250 mM NaCl), centrifugation (30 min, 17 900 rcf, 4 °C), and resuspension (in folding buffer, 30 min, shaking at 30 °C). DNA origami solutions were stored at −20 °C until use. Before use, DNA origami solutions were diluted with dilution factors adjusted differently for different sample types, typically on the order of 1:20 relative to the concentration obtained after PEG purification.

### Surface-Immobilization of DNA Origami

Liquid chambers were assembled from coverslips (22 × 22 mm^2^, no. 1.5, Marienfeld) and microscopy slides (Menzel-Gläser) using double-sided sticky tape (Scotch Transparent 665, Conrad) as a spacer. Chambers (ca. 20 μL volume) were passivated with biotinylated BSA (A8549; 1 mg/mL in buffer A, 3 min), washed with 40 μL of buffer A, and functionalized with streptavidin (S888, Thermo Fisher, 0.5 mg/mL in buffer A, 3 min). After washing with 40 μL buffer A and 40 μL buffer B, DNA origami were washed in (20 μL, in buffer B, 6 min). After incubation, unbound origami were washed out with 80 μL of buffer B. Finally, samples were washed with 40 μL of imaging solution (buffer D with imager strands and POCT oxygen scavenger) and sealed in an air-tight container with two-component epoxy glue (Toolcraft Epoxy Transparent, Conrad). The POCT oxygen scavenger consisted of 20 μg/μL catalase (P4234), 0.26 μg/μL pyranose oxidase (C40), 1 μg/μL trolox (238813), and 0.8% *w*/*w* glucose.

### Supported Lipid Bilayer (SLB) Preparation and Membrane-Tethering of DNA Origami

SLBs were formed via vesicle fusion. Lipids dissolved in chloroform were mixed in glass vials, and after solvent evaporation under N_2_ flow, the lipids were resuspended in SLB formation buffer to 4 μg/μL. The obtained large multilamellar vesicle suspensions were then sonicated (Bransonic 1510, Branson) until the solutions were clear. These small unilamellar vesicle (SUV) solutions were either used immediately or stored at −20 °C and re-sonicated before use. For fluorescence imaging of SLBs, sample chambers were assembled from cut 0.5 mL reaction tubes glued (NOA 68, Norland) onto ethanol- and water-rinsed coverslips and cured under 365 nm UV light exposure for 20 min. Immediately before use, chambers were surface-etched with oxygen plasma (30 s, 0.3 mbar, Zepto, Diener Electronics). Next, 75 μL of diluted SUV suspension (ca. 0.5 μg/μL in SLB formation buffer) were added into prewarmed (37 °C) chambers and incubated for 5 min, during which SLBs formed. After formation, SLBs were washed with 2 mL of SLB washing buffer, followed by 600 μL of buffer B. After the sample cooled to room temperature, the supernatant was replaced with 100 μL of 10 nM TEG-chol anchor oligonucleotide solution (buffer B, 3 min), followed by washing with 200 μL buffer B. Next, 100 μL of DNA origami solution was added (buffer B, 6 min), and the sample was washed with 200 μL of buffer B, followed by 200 μL of buffer D, and finally flushed twice with 200 μL of each imaging solution in buffer D with POCT. SLBs used in fluorescence experiments consisted of DOPC with 1 mol % biotinyl-cap-DOPE (both Avanti Polar Lipids) and 0.01 mol % Atto655-DOPE (ATTO-TEC). The biotin functionalization was not exploited in generating the data shown in this manuscript. SLBs for AFM imaging consisted of DOPC with 0.1 mol % Atto655-DOPE and were prepared on coverslips (22 mm diameter, no. 1, Marienfeld) in dedicated sample chambers for liquid-phase AFM (JPK). Atto655-DOPE was used to locate and quality-check membranes but not for generation of the data shown here. For preparation of SLBs for AFM, the same protocol was followed with the reagent volumes scaled up 2- to 3-fold compared to the chambers used for fluorescence imaging.

### Total Internal Reflection Fluorescence Microscopy

Fluorescence microscopy was performed at a custom inverted microscope described in detail in a previous publication.^31^ Light from a solid-state laser (561 nm, DPSS-System, MPB) was intensity-adjusted using a half-wave plate and a polarizing beam splitter (WPH05M-561 and PBS101, THORLABS). The beam passed through a refractive beam-shaping device (piShaper 6_6_VIS, AdlOptica) to create a flat illumination profile. To achieve evanescent-field illumination, the beam excentrically entered the oil immersion objective lens (100× NA 1.49 UAPON, Olympus). Fluorescence emission was collected by the same objective and filtered through suitable band-pass filters (605/64, AHF Analsentechnik) before detection on a CMOS camera (Zyla 4.2, Andor). During acquisitions, the temperature was stabilized at 23 °C (H101-CRYO-BL, Okolab), and *z*-positioning of the sample was stabilized via a piezo stage (Z-INSERT100, Piezoconcept and CRISP, ASI). The camera was operated with the open source acquisition software μManager^32^ and images were acquired with 2 × 2 pixel^2^ binning and field of view cropping to the central 700 × 700 (prebinned) pixels to achieve an effective pixel width of 130 nm and a field of view matching the circular flat illumination profile ca. 130 μm in diameter.

#### Details for Different Acquisition Strategies

##### DNA-PAINT Microscopy

DNA origami nanostructures were functionalized with 5xR1 docking sites.^25^ The imaging solution contained 1.25 nM R1_6nt_-Cy3B imager strands. Illumination intensity was set to ca. 30 μW μm^–2^. A total of 10 000 images per data set were acquired at a frame rate of 20 Hz.

##### Single Particle Tracking

DNA origami with a 20 nucleotide (nt) adapter sequence were deposited on membranes. A [TCT]_38_ “tracking handle” docking site analogous to that described by Stehr et al.^29^ was quasi-irreversibly recruited to the origami via the adapter complement: During DNA origami deposition, 10 nM tracking-handle–adapter conjugate were additionally present. To ensure a sparse subset of labeled DNA origami nanostructures suitable for SPT, a low density of tracking-handle-coupled particles was diluted in a 20-fold excess of unlabeled DNA origami particles, i.e., the same DNA origami, except without the adapter sequence. The imaging solution contained 10 nM R5_S2_8nt_-Cy3B imager strands. Illumination intensity was set to ca. 20 μW μm^–2^. A total of 10 000 images per data set were acquired at a frame rate of 20 Hz.

##### Imaging for Correlation Analysis

DNA origami nanostructures were functionalized with 5xR1 docking sites,^25^ which were quasi-irreversibly labeled through 4 min incubation with 10 nM R1_18nt_-Cy3B. The imaging solution did not contain imager strands. Connector strands were added at 250 nM immediately before start of acquisition (ca. 10 s delay, limited by speed of pipetting and closing of microscope stage incubation chamber). A total of 300 images were acquired at a frame rate of 30 Hz at each time point along the cross-linking observation. The laser was shuttered between observation time points. Illumination intensity was set to ca. 2 μW μm^–2^.

### Fluorescence Image Analysis

Processing parameters for all fluorescence experiments are listed in Table S1.

#### DNA-PAINT Microscopy

Image stacks were processed using Picasso software.^26^ Picasso Addon^7^ was used for automation. The Python software can be found on Github (https://github.com/schwille-paint). The general pipeline started with Picasso Localize to pick and localize emitters, followed by Picasso Render for drift correction (RCC). In the case of biotin/streptavidin-immobilized origami, particles were manually picked in Picasso Render, followed by automated picking of similar particles and drift correction from picked particles. The average image from many immobilized DNA origami nanostructures was created using Picasso’s Average3 module.

#### Single-Particle Tracking

The analysis pipeline started with localization in Picasso Localize as in the case of SMLM. Subsequent steps used the “SPT” package, which is also available via the above-mentioned GitHub page, for linking of localizations into tracks and mean-squared displacement analysis.

#### Correlation Analysis of Cross-Linking Kinetics

Image stacks were analyzed using a custom Python script, which is included in the Supporting Information. A detailed explanation of the analysis can be found in the Supporting Information, including a description of the simulations performed to test the accuracy of the analysis.

### Atomic Force Microscopy

Measurements were performed on a JPK Nanowizard 3. The AFM images were taken in QI (quantitative imaging) mode using BioLever Mini BL-AC40TS-C2 cantilevers (Olympus). The set point force was 0.25–0.35 nN, acquisition speed 66.2 μm s^–1^, *Z*-range 106 nm; 10 × 10 μm^2^ fields of view were acquired with a 15 nm pixel size. Images were first processed in JPKSPM Data Processing (JPK, v6.1.142) performing a line-wise second-degree polynomial leveling followed by another second-degree polynomial leveling with limited data range (0% lower limit, 70% upper limit). Subsequent plane leveling, third-degree polynomial row alignment and scar correction were performed in Gwyddion (v2.58, http://gwyddion.net/).

## Results and Discussion

### Simple DNA Origami Design for Cross-Linking Studies

To study DNA origami cross-linking, we first designed a suitable monomer structure. We reasoned that the use of a well-characterized modular structure would be most convenient and thus opted for a flat rectangular grid origami used in a number of previous single-molecule fluorescence studies.^7,25,29,33,34^ On this monomer structure, we arranged 36 DNA-PAINT docking sites in the shape of an arrow. This design challenges the resolution in DNA-PAINT imaging and allows reading out the orientation of the origami on the surface (Figure 1). DNA-PAINT imaging of individual DNA origami particles immobilized on a glass surface via biotin–streptavidin anchoring indeed revealed the expected arrow pattern with high yield (Figure 1c).

**Figure 2:**
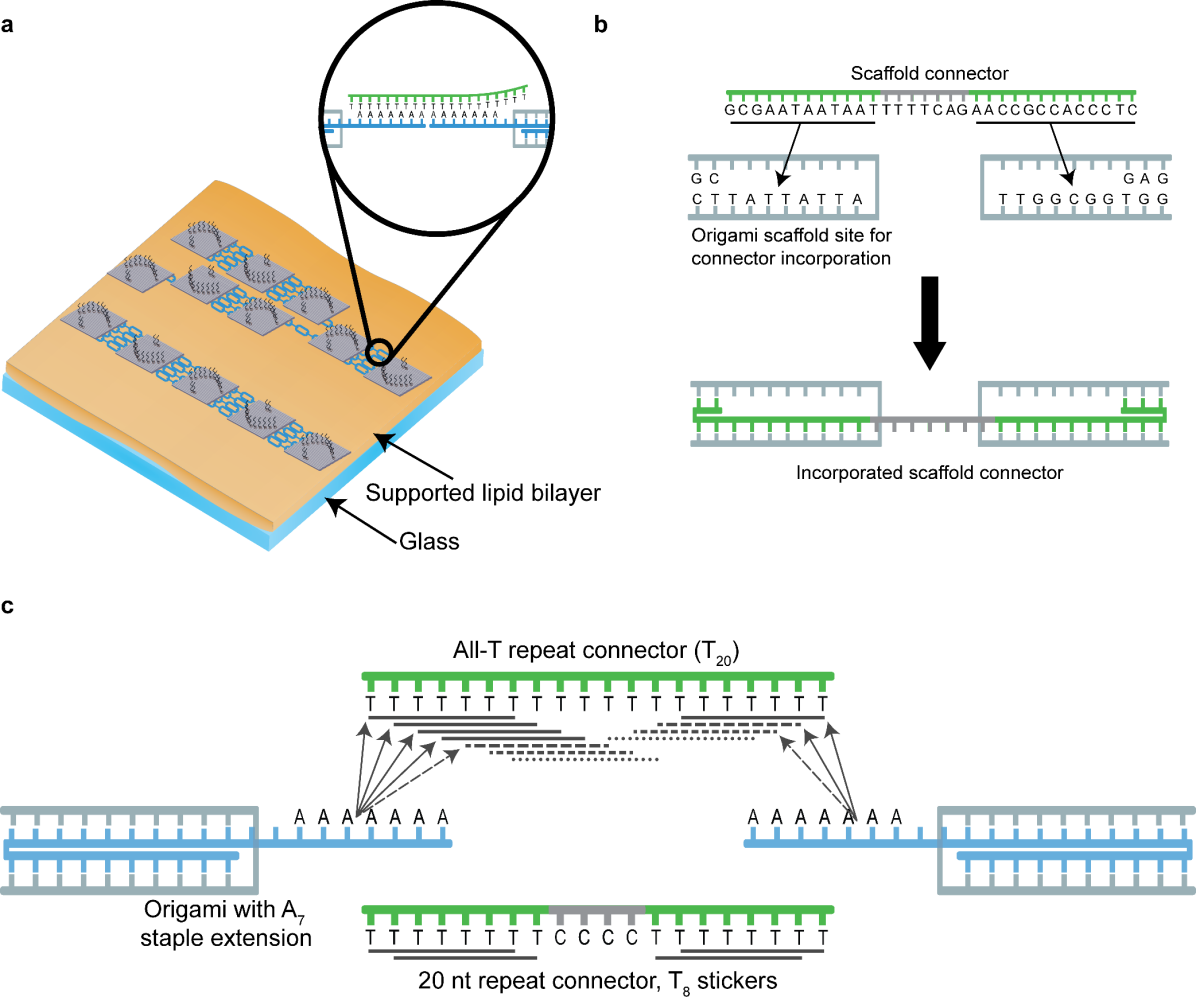
Schematic of DNA origami cross-linking kinetics on membranes. (a) Cross-linking geometry. Cross-linking sites are distributed on the DNA origami such that linear assemblies are expected, but with repeat connectors branching is also possible. (b) Scaffold connectors directly bind scaffold loops of two DNA origami particle, yielding highly site-specific assembly. (c) Repeat connectors bind the DNA origami indirectly via A_7_ staple extensions. Depending on the design of the connector strand, many binding reading frames are available for the A_7_.

We then functionalized the “bottom” side of the origami structure with staple extensions to bind it to supported lipid bilayer membranes (SLBs) via complementary TEG-chol-coupled oligonucleotides. Only two opposing lateral edges of the DNA origami were further functionalized for cross-linking into higher-order assemblies, aiming for linear chains rather than tilings, as the latter might be more difficult to distinuish from unspecific clustering (Figure 2a). In all cross-linking experiments described in this manuscript, each DNA origami edge participating in the association was designed to bind four connector strands. The DNA origami design exposes no blunt ends of DNA duplexes to avoid uncontrolled association via base stacking. Figure 2 gives a schematic summary of the DNA origami cross-linking strategies. One strategy that we employed has been frequently reported before.^16,18,23^ Here, DNA origami nanostructures are cross-linked via connector strands that are essentially staple strands which incorporate into both monomers simultaneously (Figure 2b). For concision, we will call these “scaffold connectors”. The other strategy is to incorporate modified staples into the DNA origami that carry extensions for indirect binding of connector strands to the DNA origami. We reasoned that DNA origami superstructure assembly could be accelerated through a connector strand design analogous to the above-mentioned high-on-rate docking site design^25,29,33^ used for example in DNA-PAINT, i.e., the use of low-complexity sequences to increase the effective association rate (Figure 2c). We opted for short stretches of a single nucleotide species, specifically A_7_ as an extreme case of such a low-complexity sequence. The connector strands were simply oligo-T sequences. These connector strands will be referred to as “repeat connectors”. We note that we did not optimize our structure for highly specific assembly geometries. Instead, we aimed for a simple system that would serve as a model system for characterizing the assembly process itself. Thus, a stochastically assembling design was chosen in which also the shape of the formed structures would reveal the action of the connector strands in super-resolution imaging. With the basic origami design and cross-linking strategies at hand, we proceeded to create higher-order DNA origami assemblies on fluid membranes.

### Repeat Connectors Are a Viable Option for Superstructure Assembly

**Figure 3:**
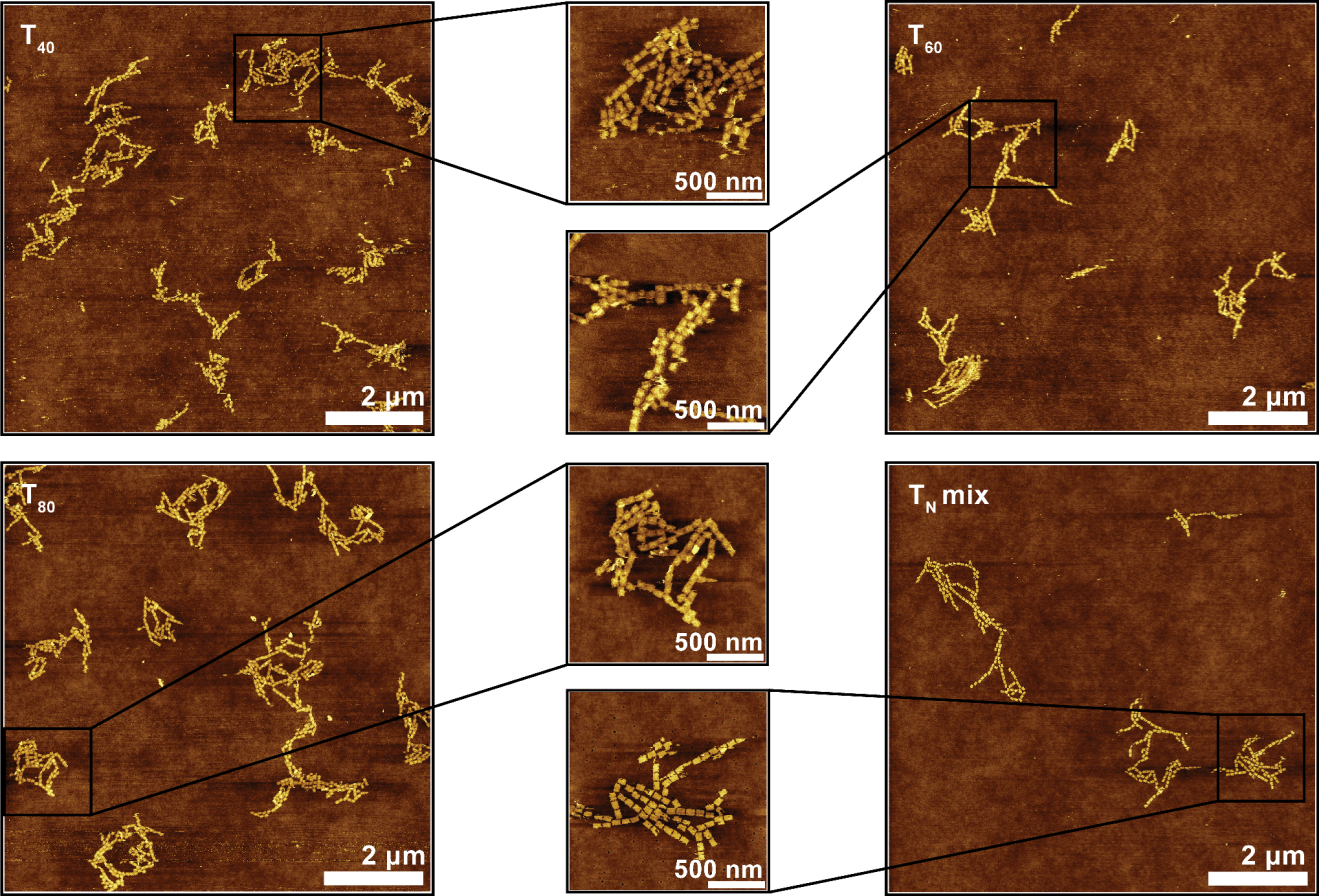
AFM characterization of DNA origami superstructures, showing conditions which yielded high-quality images. Additional conditions are shown in Figure S3. All images were acquired after 2 h incubation with 250 nM of the specified connector strand. The T_*N*_ mix is 50 nM each T_14_, T_20_, T_40_, T_60_, and T_80_. The color-coded height scale in all panels is 6 nm.

We first characterized the structures of our cross-linked DNA origami structures using AFM to confirm the possibility of forming superstructures with desired geometry using repeat connectors. For AFM imaging, we prepared DNA origami samples on fluid SLBs and cross-linked them for 2 h using all-T repeat connectors of different lengths (T_14_, T_20_, T_40_, T_60_, T_80_, or a mixture of all of these referred to as T_*N*_ mix). Before imaging, we exchanged the buffer, increasing the Mg^2+^ concentration from 7.5 to 40 mM to decrease mobility of the preformed structures for better AFM image quality. When using repeat connectors, ≥40 nt in length, high-quality images showing the expected formation of extended filaments were obtained which agree with the linear assembly geometry dictated by design (compare Figures 3 and 2). However, we saw hardly any differences between different lengths ≥ 40 nt. Small oligomers formed by shorter repeat connector strands yielded lower quality images, suggesting that these led to hardly any superstructure formation within 2 h. In fact, the structures that we obtained with repeat connectors rather looked like unspecific association due to the high Mg^2+^ concentration (Figure S3). We did see some lateral assembly as well: As all cross-linking staple extensions have the same A_7_ sequence and only differ by orientation of 3′- or 5′-ends, there is no strict specificity regarding the orientation of neighboring DNA origami monomers within the superstructure. This allows branching of linear assemblies, which leads to the formation of the observed 2-dimensional superstructures. We observed this branching somewhat less frequently when using scaffold connectors, which are site-specific in their binding to DNA origami and thus suppress branching (Figure S3). The presence of some branching even in this setting suggests Mg^2+^ unspecific association. Overall, the AFM data suggests that using long repeat connectors allows to cross-link DNA origami superstructures efficiently, albeit with trade-offs in specificity. However, there was no obvious difference between the different repeat connectors that efficiently cross-linked the DNA origami structures. In our AFM experiments, pushing of DNA origami structures by the AFM tip forced us to strongly increase the Mg^2+^ concentration, which led to unspecific association. Thus, at least with lengths ≥40 nt, repeat connectors do facilitate formation of DNA origami superstructures. To characterize the structures in more detail under origami-typical buffer conditions, we employed single-molecule fluorescence imaging.

### Repeat Connectors Form Stable Superstructures Faster than Scaffold Connectors

Before acquiring super-resolution images of our samples, we used SPT to characterize particle mobility prior to cross-linking in the imaging buffer used for all following fluorescence microscopy experiments, containing 7.5 mM Mg^2+^ and 140 mM Na^+^. SPT showed that our TEG-chol-anchored DNA origami particles diffused freely on the SLBs with a diffusion coefficient of ca. 0.2 μm^2^ s^–1^ (Figure S4). However, upon addition of connector strands, we observed a strong decrease in mobility, indicating superstructure formation. A large fraction of particles was practically immobilized 30 min after addition of a mixture of oligo-T connector strands to A_7_-functionalized origami (Figure S5). We reasoned that these may in fact be sufficiently immobilized for DNA-PAINT-based structural characterization using an accelerated acquisition protocol following Strauss and Jungmann,^25^ which reduces the acquisition time to ca. 8 min. SMLM has been successfully applied to samples with slow but non-negligible motion such as live cells before, albeit with trade-offs between acquisition time and resolution.^35,36^

**Figure 4:**
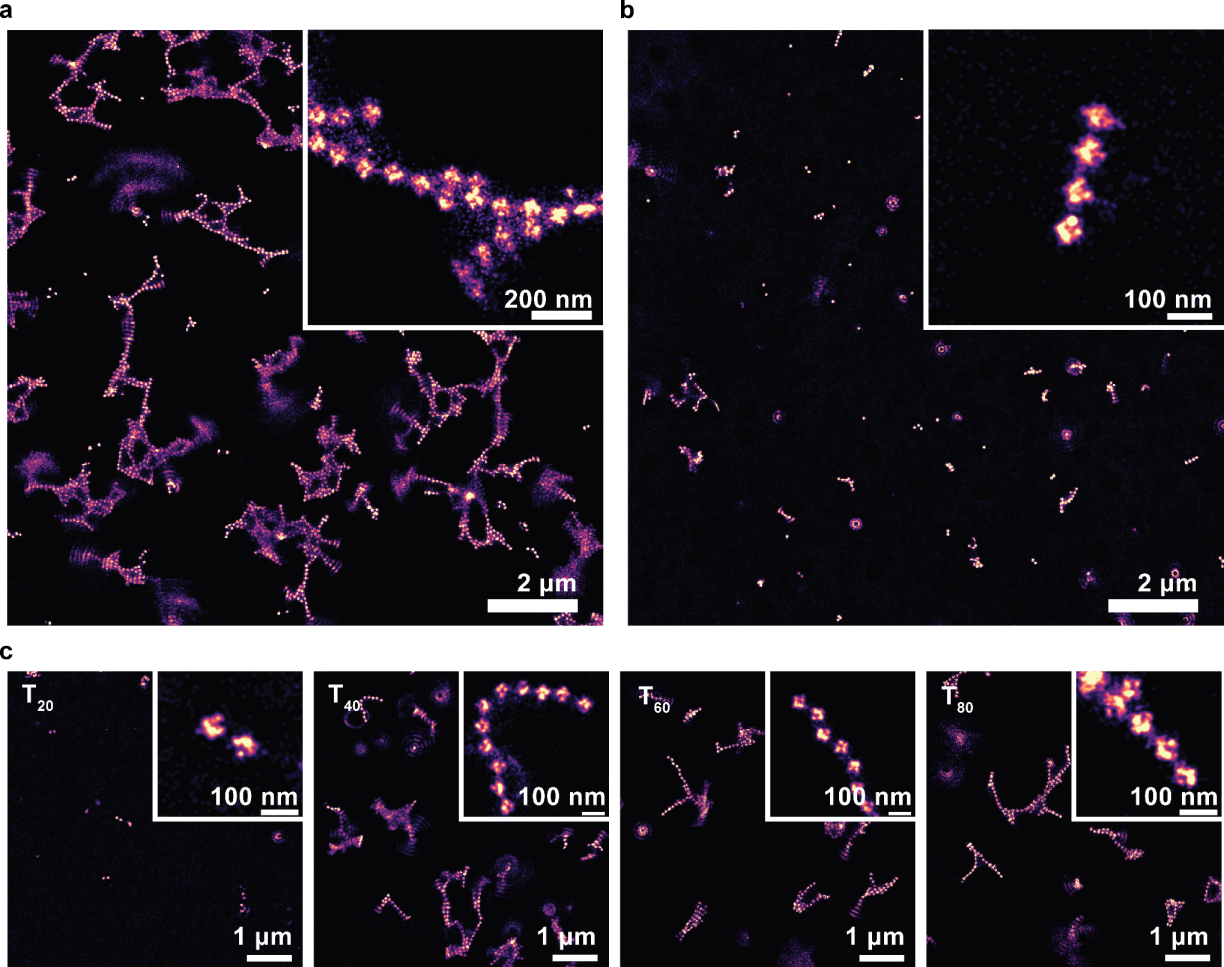
DNA-PAINT characterization of DNA origami superstructures cross-linked with different connector strands. (a) T_*N*_ repeat connector mix containing T_14_, T_20_, T_40_, T_60_, and T_80_ at 50 nM each (30 min incubation). (b) Scaffold connectors (250 nM total concentration, 20 h). (c) Individual repeat connectors (250 nM, 30 min).

Even with that accelerated acquisition, we were unable to resolve any structures in DNA-PAINT imaging without cross-linking (Figure S6a). However, we were able to resolve large DNA origami superstructures on the membrane after cross-linking for only 30 min with the T_*N*_ repeat connector mixture (Figure 4a). Notably, in all our AFM and DNA-PAINT experiments, the connector strand solution had been replaced with connector strand-free imaging buffer before acquisition. This means that the observed assemblies were rather stable and did not undergo rapid dissociation/reassociation dynamics and, in particular, that the assemblies were not dependent on stabilization by the high Mg^2+^ concentration in the AFM imaging buffer. This confirms that the use of short A_7_ sticker sequences combined with multivalent cooperative binding is sufficient for association of stable superstructures. In fact, the branching of oligomers seen in AFM and confirmed by SMLM suggests that our A_7_ cross-linking extensions are too long for efficient “self-healing” of association sites into “ideal” association geometries.^12,37^ We saw similar results when using scaffold connectors, but much longer incubation times were needed before high-quality imaging was possible: Compare Figure 4b acquired after 20 h to Figure S7 acquired after 2 h. This is in line with previous publications using scaffold connectors to cross-link DNA origami into 2D systems.^18,23^ Each scaffold connector first needs to bind to its unique binding site on a DNA origami nanoparticle and then to the appropriate binding site on a second particle, requiring the DNA origami monomers to collide in the correct mutual orientation. Even after 20 h, only rather small assemblies were found. Thus, repeat connectors allowed assembly within less than 1 h, while scaffold connectors seemed quite unsatisfying regarding throughput of the experiment.

Although the image resolution in DNA-PAINT on membranes was lower than that in the image of origami directly immobilized on glass, we achieved resolution down to the 10 nm scale even on membranes. The resolution was limited by residual motion on the time scale of the acquisition, as demonstrated by the blurred clouds of localizations in various positions of the image. The orientation of some DNA origami monomers within the context of the superstructures was visible in the SMLM images, giving access to some information about the geometry in association. When repeat connectors are used, both parallel and antiparallel arrow orientations in neighboring particles are seen, which is obviously another consequence of the lack of site specificity in repeat connector binding. This is in stark contrast to the images obtained using scaffold connectors, which yield assemblies specifically with parallel orientation (Figure 4b). Notably, DNA-PAINT imaging of DNA origami deposited in a 3-fold higher density, but not exposed to connector strands, yielded low-resolution images of very different structures (Figure S6b). This confirms that despite the compromises in association geometry specificity when using repeat connectors, the retrieved superstructures are products of hybridization-based, connector strand-dependent association.

Finally, we compared superstructures formed by different lengths of all-T connector strands using DNA-PAINT imaging (Figure 4c). T_14_ (not shown) or T_20_ repeat connectors showed almost no cross-linking within 30 min, supporting the idea that assembly seen with AFM was mostly unspecific due to the high Mg^2+^ concentration. As in AFM, we saw little difference between the different all-T connectors of lengths ≥ 40 nt. From our DNA-PAINT experiments, we could thus confirm the connector strand-driven association of our DNA origami superstructures, and that long repeat connectors yield faster assembly than scaffold connectors. Motivated by these findings, we decided to characterize more quantitatively the differences between assembly kinetics of scaffold and repeat connectors, in order to obtain a mechanistic understanding of these differences.

### Quantification and Mechanisms of Assembly Acceleration

**Figure 5:**
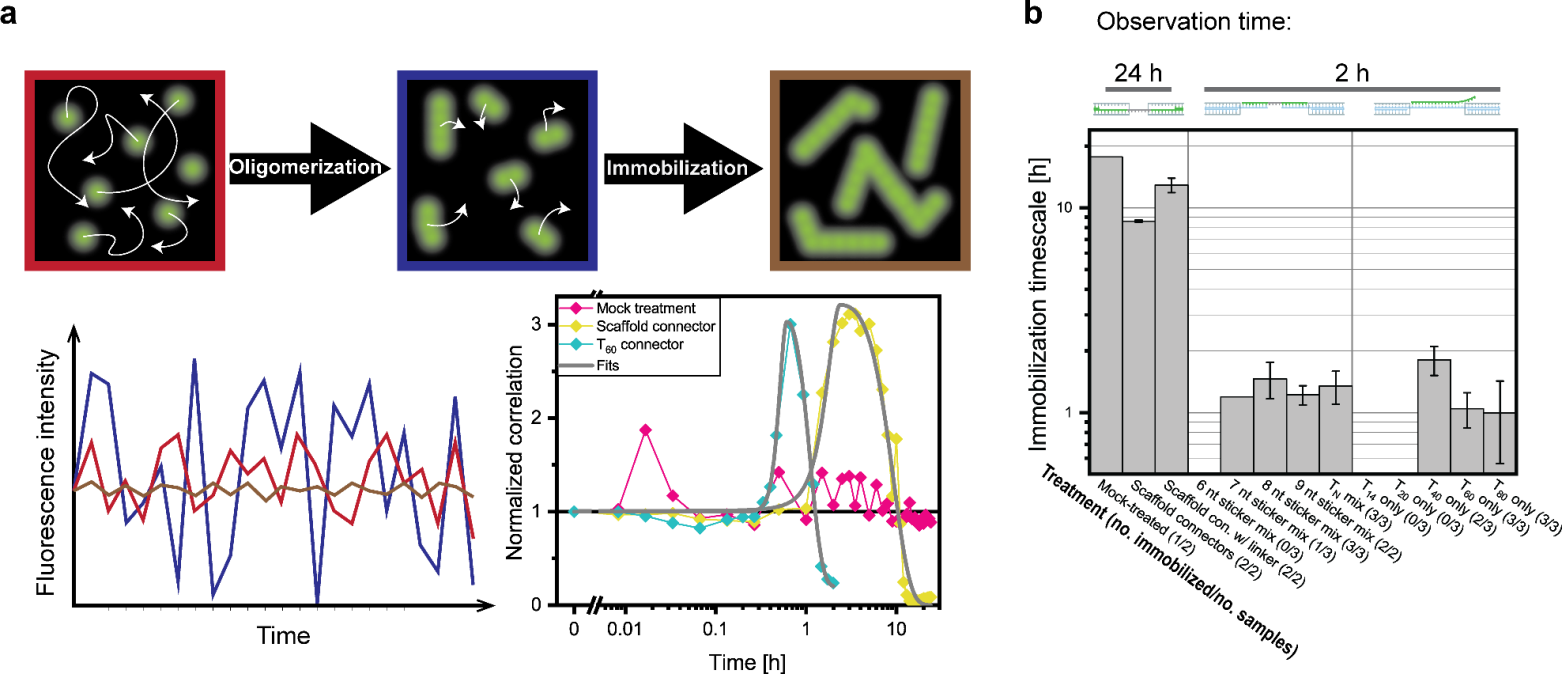
Correlation analysis of cross-linking kinetics. (a) Illustration and example data of correlation analysis. At the beginning of the experiments, monomers diffuse rapidly, creating moderate fluorescence fluctuations (red image and fluorescence intensity trace). As oligomerization begins, effectively fewer brighter particles are observed, increasing fluctuation amplitudes at unchanged average intensity (blue). As oligomerization progresses, yielding large, immobile particles, fluctuations become negligible (brown). The time traces of correlation parameter change show two examples of traces quite clearly undergoing these phases within observation time, and a buffer-treated negative control. (b) Kinetics of DNA origami higher-order assembly measured through image correlation analysis (mean ± s.d.). See the main text for details about the different conditions. Numbers in parentheses refer to the number of data sets for which an assembly time scale could be fitted compared to the number of data sets acquired for this condition. One of the mock-treated samples did show clear immobilization, which we attribute to unspecific sample degradation.

In the next experiments, we set out to determine characteristic time scales for DNA origami higher-order assembly under different conditions. We opted for an image correlation analysis-based read-out of oligomerization (see Supplementary Note and Figure S1). The calculated correlation parameter, reporting the amplitude of temporal fluorescence fluctuations, increases as the particles associate into higher-order assemblies: Fluorescence fluctuations are larger when few bright particles diffuse through a pixel than many dim ones do. Later, the correlation parameter falls to zero or a low baseline value, as the assemblies become so large that they are essentially immobile during the 10 s observation: Immobile particles yield an approximately constant signal over time (Figure 5a). The correlation analysis was found to be sensitive to oligomerization and immobilization in simulations of different ratios of mono- and oligomers (Figure S2). Additional advantages for long-term observation of the overall evolution of the sample are lower illumination intensities and the fact that in contrast to SMLM and AFM, this analysis captures the entire ensemble of particles rather than selectively showing immobile assemblies. Thus, image correlation analysis provided a convenient aggregate readout for higher-order assembly kinetics, from which we derived characteristic time scales of immobilization as a surrogate for assembly of DNA origami superstructures (Figure 5b). For these experiments, the spatial arrangement of docking sites previously used for DNA-PAINT plays no role (ca. 50 nm pattern width vs ca. 200 nm spatial resolution). Instead, we created bright particles through quasi-irreversible binding of multiple long R1_18nt_-Cy3B imager strands to the full length of the docking site.^38^ We systematically compared cross-linking by a variety of connector strands under otherwise constant conditions. These included the previously used scaffold connectors with and without short flexible linkers between the binding sites and all-T repeat connectors of lengths 14, 20, 40, 60, and 80 nt. In addition, we included mixtures of repeat connectors of all lengths, but with inserted oligo-C spacers that do not bind the oligo-A extensions, thus tuning the “sticker” length (i.e., number of binding reading frames) without changing the overall length of the connector strands (Figure 2c). The results are compiled in Figure 5b for comparison, but they will now be discussed sequentially.

Assembly kinetics were observed following addition of connector strands for either 24 h (scaffold connectors and negative controls) or 2 h (repeat connectors). Confirming the findings from DNA-PAINT imaging, very long incubation times in the order of 10 h were needed to create fully assembled structures using scaffold connectors. Adding a short flexible linker sequence to the scaffold connectors did not strongly affect the association kinetics. If anything, it slowed down association, which may be explained by the findings of Zenk et al.^20^ that larger flexibility of connector binding sites can be detrimental to association.

We then characterized the repeat connectors with total lengths of 14, 20, 40, 60, and 80 nt. First, we looked at cross-linking kinetics for mixtures of repeat connectors with internal oligo-C stretches and terminal oligo-T stickers. Oligo-T sticker lengths varied from 6 nt (shorter than the A_7_ docking site) to 9 nt (three binding reading frames). Using repeat connector mixtures for cross-linking, we saw a strong acceleration in association kinetics for sticker lengths of ≥7 nt. Within the 2 h acquisition time, we did not see any notable changes in the fluctuation data for 6 nt stickers, and for 7 nt stickers, only one out of three samples showed immobilization. Increasing the oligo-T sticker length at the end of the repeat connectors to 8 or 9 nt yielded robust assembly within <2 h, demonstrating the desired acceleration. These sticker lengths offer 2 or 3 reading frames for the A_7_ binding partner, respectively, meaning that the data is entirely consistent with our idea of multiple reading frames accelerating binding. Another cause for acceleration is the same effect that is the cause for the reduced orientation specificity observed by nanoscale imaging: Repeat connectors can bind various positions on DNA origami nanoparticles, reproducing the effect of multivalent binding previously reported.^20,24^ Time-resolved analysis of cross-linking kinetics thus confirms an order-of-magnitude acceleration in assembly dynamics by using our repeat connector strategy, as compared to our scaffold connector strategy.

Interestingly, no further acceleration of superstructure assembly was seen by using a mixture of all-T connector strands of different lenths (“T_N_ mix” in Figure 5b) compared to those with 8 or 9 nt stickers. We hoped to find an explanation for this effect by comparing different lengths of all-T connector strands. The longer all-T repeat connectors accelerated assembly compared to shorter ones. While Zenk et al.^20^ argued that increasing connector strand flexibility (i.e., length) can be detrimental to binding, here the increased length comes with an increase in the number of binding sites. We did not see immobilization within 2 h using T_14_ or T_20_ connectors. This suggests an explanation for the fact that using a mix of different all-T connectors did not further accelerate assembly relative to connectors with 9 nt stickers: The inefficient T_14,20_ connectors likely competed with the more efficient T_40,60,80_ connectors. The low efficiency of T_14,20_ connectors may be explained by the fact that their short sequences cause A_7_ docking sites to compete for overlapping binding sites on the same connector strand, which is clearly detrimental for cross-linking. This competition is suppressed in repeat connectors with internal oligo-C stretches and less relevant in long all-T ones.

Obviously, by comparing scaffold connectors to repeat connectors only consisting of oligo-T stretches, we looked at two extremes in a broad spectrum of thinkable cross-linker designs: one entirely optimized for assembly speed and the other entirely for specificity. Intermediate strategies would allow different trade-offs between these parameters. For example, one could combine oligo-A staple strand extensions with oligo-G staple extensions, creating two orthogonal cross-linking systems. These could also be combined through connectors concatenating oligo-T stretches and oligo-C stretches to link an oligo-A functionalized DNA origami face to an oligo-G functionalized one. This would increase specificity in assembly geometry, unlikely to result in antiparallel association of our DNA origami monomers. Repeats of 2 or 3 nt sequence motifs further increase the number of orthogonal motifs available for cross-linking,^25^ but the number of binding reading frames will decrease rapidly with increasing motif length. Notably, such 2 nt motifs, albeit without repeats, were used previously to create very large DNA origami superstructures^12^ with high specificity in assembly geometry. However, this specific formation of large structures required a multistep assembly that is slow and is not easily transferred to the *in situ* assembly in which we were interested.

Finally, an additional mechanism that likely contributes to the acceleration of binding using low-complexity sequences is the absence of internal hairpins from oligo-T or A_7_ sequences. Hairpin formation can strongly reduce effective on-rates.^33,34^ Due to sequence constraints from direct binding to the scaffold strand, hairpin formation could not be abolished completely in the design of the scaffold connectors used in this study according to the prediction by NUPACK.^39^ One might thus consider high-complexity, yet hairpin-free, docking site extensions. While sequence design will become very challenging with increasing numbers of desired orthogonal sequences and the speed gain will likely remain modest compared to what our work demonstrates, such an approach remains highly attractive regarding specificity. In any case, our recommendation for designing rapidly cross-linking sequences for DNA origami superstructures is to avoid direct binding of connector strands to the scaffold and instead use staple extensions, designed with the lowest possible sequence complexity sufficient to ensure the required specificity.

## Conclusions

In this work, we compared different design features to optimize assembly kinetics of higher-order DNA origami structures. A significant acceleration was achieved by cross-linking DNA origami indirectly via low sequence complexity connector strands binding to staple strand extensions, instead of direct binding of high-complexity sequences to loops in the scaffold DNA. We postulate two effects to contribute to the increased speed: The presence of multiple binding reading frames increases the effective local concentration of binding sites, and thus the effective association rate, and the used low-complexity sequences prevent the formation of hairpins. Using modifications of the strategy will allow multiple orthogonal sequences, increasing association specificity, with some trade-off in experimental throughput. This quite simple and generic approach to accelerate DNA origami superstructure assembly should prove useful to increase throughput of experiments in the field and to benefit experiments that require time-controlled assembly.

## Supporting Information Available

Methods, image correlation analysis workflow, simulations to assess sensitivity of image correlation analysis to oligomerization and immobilization, AFM results, example of single particle tracking results for monomeric DNA origami particles on DOPC SLBs, DNA-PAINT imaging of non-cross-linked and incompletely cross-linked DNA origami nanostructures, image analysis parameters, oligonucleotide sequences for DNA origami design, additional references (PDF)
MATLAB and Python scripts used for simulation and analysis of cross-linking kinetics (ZIP)

## Abbreviations

AFM: Atomic force microscopy
DNA-PAINT: DNA point accumulation for imaging in nanoscale topography
FCS: Fluorescence correlation spectroscopy
SLB: Supported lipid bilayer
SMLM: Single-molecule localization microscopy
SPT: Single-particle tracking
ssDNA: Single-stranded DNA
SUV: Small unilamellar vesicle
TEG-chol: Tetraethyleneglycol–cholesterol
