# Supplementary material for: Design Features to Accelerate the Higher-Order Assembly of DNA Origami on Membranes
Source: J Phys Chem B. 2021 Nov 24;125(48):13181–91. doi: 10.1021/acs.jpcb.1c07694 (PMC8667037; doi:10.1021/acs.jpcb.1c07694)
Supplement: Supplementary file 1 — jp1c07694_si_002.pdf [file jp1c07694_si_002.pdf]

Supplementary Information
Design Features to Accelerate the Higher-Order Assembly of DNA Origami on Membranes

Yusuf Qutbuddin,[†,¶] Jan-Hagen Krohn,[†,‡,¶] Gereon A. Brüggenthies,[†] Johannes Stein,[†] Svetozar Gavrilovic,[†] Florian Stehr,[†] and Petra Schwille[*,†]

†*Department of Cellular and Molecular Biophysics, Max Planck Institute of Biochemistry, Am Klopferspitz 18, D-82152 Martinsried, Germany*

‡*Exzellenzcluster ORIGINS, Boltzmannstr. 2, D-85748 Garching, Germany*

¶*Equal contribution*

E-mail: schwille@biochem.mpg.de

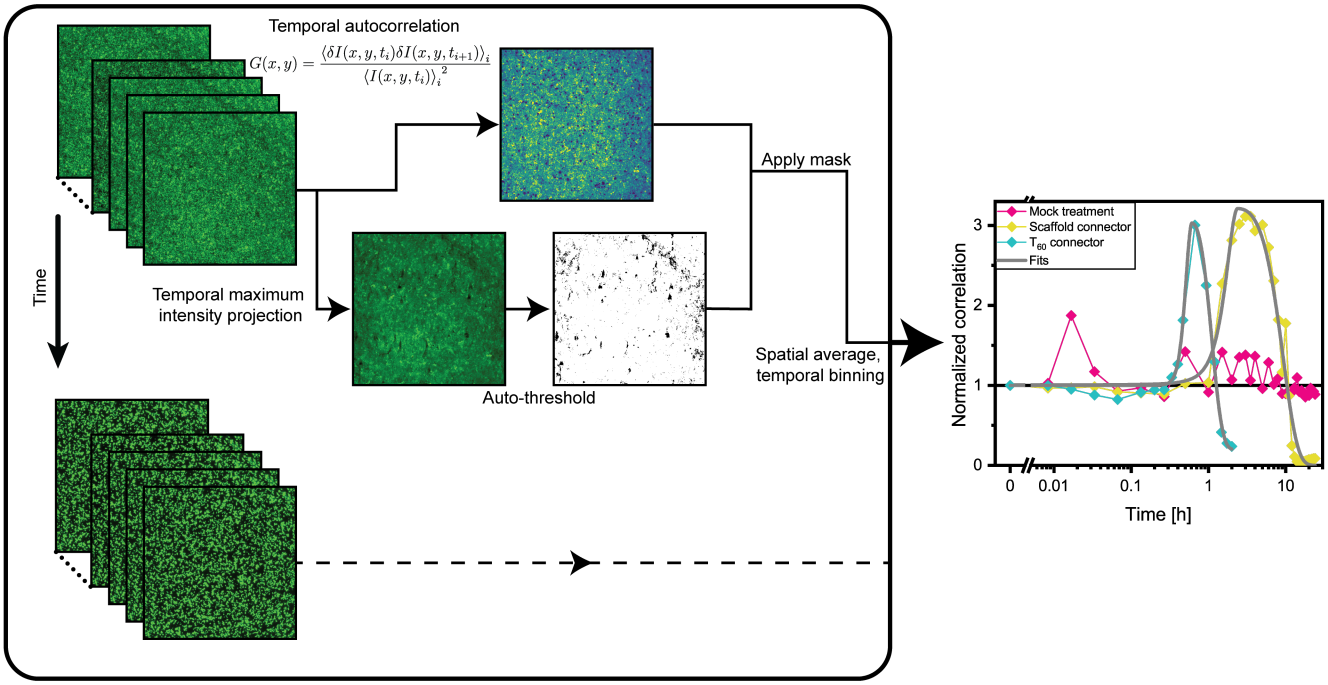

Figure S1: Image correlation analysis workflow. An acquisition consists of a series of blocks with a few hundred frames each. From each acquisition block, a correlation image and a thresholded mask (e.g., to remove membrane defects from the analysis) are calculated, and from these a single average correlation parameter is calculated, which serves as an estimate of the autocorrelation amplitude. This value is calculated independently for each bock in an acquisition, and the (normalized) trace of correlation amplitude over time is fitted to extract a characteristic timescale of immobilization. The right panel shows three example traces from different conditions with asymmetric Gaussian fits.

In this section, will first describe the workflow for our image correlation analysis, followed by an explanation of the underlying concept. Our correlation analysis is based on the following autocorrelation expression:

$$G(x, y) = \frac{\langle \delta I(x, y, t_i)\delta I(x, y, t_{i+1})\rangle_i}{\langle I(x, y, t_i)\rangle_i^{\,2}} \tag{S1}$$

with frame-wise pixel greyscale $I(x, y, t_i)$, greyscale fluctuation $\delta I(x, y, t_i) = I(x, y, t_i) - \langle I(x, y, t_i)\rangle_i$ and $\langle ...\rangle_i$ as averaging over parameter i, here (discrete) time. This expression was applied to the time-dependent greyscale of every image pixel within each 10 s acquisition block. Further, a temporal maximum intensity projection (MIP) of the 10 s image stack was calculated and automatically thresholded to eliminate positions in which no particles

were present throughout the acquisition block, e.g., membrane defects. Autocorrelation values $G(x, y)$ from pixels within the mask from MIP thresholding were averaged to yield a single correlation value $G(j)$ characterizing the entire acquisition block j. The correlation values for a given experiment consisting of multiple 10 s blocks were then normalized as $G_n(j) = G(j)/G(j = 1)$. To extract characteristic timescales, the retrieved curves were fitted in OriginPro (2019b, OriginLab, unweighted Levenberg-Marquardt fit) with an asymmetric Gaussian function:

$$G_n(j) = \begin{cases} 1 + A_1 \cdot e^{[\frac{-ln2(t_j - t_0)^2}{T^2_{\frac{1}{2},rise}}]} & for\ t_j \leq t_0 \\ G_\infty + A_2 \cdot e^{[\frac{-ln2(t_j - t_0)^2}{T^2_{\frac{1}{2},drop}}]} & for\ t_j > t_0 \end{cases} \tag{S2}$$

with $A_2 = 1 + A_1 - G_\infty$. A_1 and G_∞ characterize amplitude and asymptote of $G_n(j)$, respectively, and t_0 is the peak position. $T_{\frac{1}{2},rise}$ and $T_{\frac{1}{2},drop}$ are peak half-widths for rising and falling edge of the correlation change. Reported values in Figure 5b are $t_0 + T_{\frac{1}{2},drop}$ as overall timescale of immobilization (i.e., drop in correlation). The use of a Gaussian function is rather arbitrary, motivated by its simplicity and the fact that no other model was found to yield notably better fits to the shapes of the traces.

The correlation analysis used to characterize cross-linking kinetics is a simplified implementation of the concept of fluorescence correlation spectroscopy (FCS), in particular temporal image correlation spectroscopy (TICS).[1] We will not review that theory in detail but only explain how the correlation parameter shown in Figure 5 relates to oligomerization and immobilization. Details can be found in the FCS literature, e.g. refs. 2, 3, and 4. In FCS/TICS, fluorescence intensity time traces are analyzed through an expression closly resembling Equation S1:

$$G(\tau) = \frac{\langle \delta F(t) \delta F(t + \tau) \rangle_t}{\langle F(t) \rangle_t^2} \tag{S3}$$

with time-resolved fluorescence signal $F(t)$ and correlation lag time τ. The autocorrelation parameter $G(x, y)$ determined in our image correlation analysis serves as an estimate of the

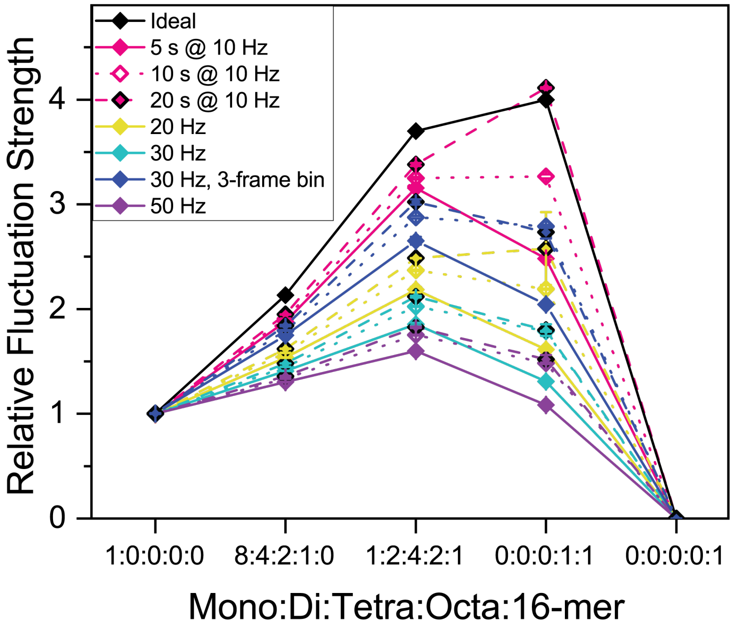

Figure S2: Simulations to assess sensitivity of image correlation analysis to oligomerization and immobilization. 16-mers are immobile in these simulations, their increasing fraction decreases the fluctuation amplitude. Symbol types and linestyles indicate acquisition block duration, colors indicate frame rate. Black line is the prediction according to Equation S6.

correlation amplitude G_0, which in simple settings directly yields the number of particles in the observation volume:

$$G_0 = \lim_{\tau \to 0} G(\tau) = \frac{1}{\langle N(t) \rangle_t} \tag{S4}$$

which is true if one assumes the number of particles in the observation volume over time $N(t)$ to be Poisson-distributed and $F(t) = \varepsilon N(t)$, i.e. all particles to be described by the same brightness parameter ε. This relation is true independent of the specific value of ε, and independent of the diffusion coefficient.

Now consider a mixture of fluorescent signals from multiple species A with distinct brightnesses, e.g., different oligomerization states, as well as a static signal contribution from an immobilized particle fraction S:

$$F(t) = \varepsilon_S N_S + \sum_A \varepsilon_A N_A \tag{S5}$$

Assuming a Poisson-distributed $N_A(t)$ as before and that the different species do not interact, G_0 becomes:

$$G_0 = \frac{\sum_A {\varepsilon_S}^2 \langle N_A(t) \rangle_t}{\left[\varepsilon_S N_S + \sum_A \varepsilon_A \left\langle N_A(t) \right\rangle\ \right]^2} \tag{S6}$$

Fluctuations in the signal from S only originate from shot noise, which is temporally uncorrelated and thus eliminated from the analysis by estimating G_0 from nonzero correlation times (extrapolation of $\lim_{\tau \to 0} G(\tau)$ in FCS, one-frame temporal lag in our analysis). As a consequence, immobilization of particles manifests as a drop in G_0: S contributes only to the denominator of the correlation function. Thus, increasing the number of immobile particles (fraction S) while decreasing the number of mobile particles (fractions A) will decrease the numerator of G_0, but leave the denominator unchanged. On the other hand, G_0 increases with progress of oligomerization. To understand this, one has to remember that producing e.g. one dimer particle consumes two monomer particles. This means that with progress of association, the overall number of particles will decrease. Further, the different oligomer species contribute to G_0 with different weights given by their squared brightness ${\varepsilon_A}^2$, which increases with higher oligomer stoichiometry. Therefore, a moderate number of large oligomers will overwhelm the correlation signal from a large number of monomers or small oligomers: The correlation amplitude will indicate a small overall particle number (i.e., large G_0).

We employed simple Monte-Carlo simulations to test the sensitivity of this analysis for oligomerization and immobilzation. Simulations of near-single-molecule imaging acquisitions were performed using custom software written in MATLAB (R2020b, The MathWorks). A strongly simplified system that yields data similar to that experimentally observed was simulated: A constant number of $5 \cdot 10^5$ particles were split into user-defined factions of monomers, dimers, tetramers, octamers, and 16-mers. The simulated field of view was equal in size to that of the experimental acquisitions expanded in each direction by 10 %. Particles were placed with random positions and orientations in the simulated area. Then, a random walk simulation with periodic boundary condition was performed. In each one-frame time step,

particles were displaced by Gaussian-distributed random distances in both x and y, each with 0 mean and standard deviation $\sqrt{2D[pixel^2 \cdot frame^{-1}]}$. The diffusion coefficient of monomers was assumed to be 0.1 μm^2 s^{-1}, similar to the experimentally observed value (Figure S4). Oligomer diffusion coefficients were assumed to be the monomer diffusion coefficient divided by the oligomer stoichiometry as a simple model of decreasing mobility with increasing oligomer size. 16-mers were fully immobile, representing the immobilized fraction. Rotational motion was neglected. After completion of the random walk simulation, localizations in the added 10 % edge were discarded to create a field of view equal comparable to the experiments. From the frame-wise center of mass positions and the orientations, images of linear oligomers were then generated with Gaussian-shaped point spread functions with resolution similar to the experiments. Poisson-distributed photon shot noise and realistic values for Poisson-distributed fluorescent background, camera offset and Gaussian-distributed readout noise were applied to create image stacks comparable to the experimental ones. Simulations were performed in blocks of 20 s, and each condition (oligomer fractions, frame rate) was simulated three times. For simulations of shorter acquisitions times, the 20 s simulations were cropped, so that finally for 20 s acquisitions three simulations per condition were analyzed, for 10 s acquisitions six, and for 5 s acquisitions twelve.

Comparing the simulation results to predictions according to Equation S6 (Figure S2), we see that with 10 Hz frame rate and 20 s observation time, we almost perfectly retrieve the theoretical values. Lower exposure times per frame and shorter acquisitions seem to yield data less sensitive particularly to association, presumably mainly due to signal-to-noise ratio limitations. Binning three successive frames of the 30 Hz simulations into a single frame (i.e., reducing time resolution to 100 ms without otherwise changing the data content) restores some of the sensitivity, again suggesting that signal-to-noise ratio is the main limitation. As our experimental data had been acquired at 30 Hz frame rate with 300 frames (10 s) blocks, we decided to post-bin our experimental data the same way.

Note that increase of the correlation amplitude due to oligomerization and its decrease

due to immobilization can happen at the same time, and the correlation amplitude G_0 (or $G(x,y)$) alone is not sufficient to dissect them. Even if one constrains the oligomer brightness to increase linearly with stoichiometry (i.e., $\varepsilon_A = A\varepsilon_1$), the system remains underdefined for extraction of $\langle N_A(t)\rangle_t$ for any multi-species mixture. To be able to extract these values, one would have to introduce further of assumptions about the association process to link $G(x,y)$ over successive observations. Alternatively, for data with high signal-to-noise ratio, higher-order correlation analysis might be a viable option.[5,6] We considered these more advanced methods to be beyond the scope of this manuscript, especially considering that for our experimental system we expect rather complicated dynamics including significant effects from non-negligible particle size compared to the optical resolution. Instead, we simply fitted the traces with an asymmetric Gaussian to determine half-rise/half-decay times (Figure S1).

Supporting Figures

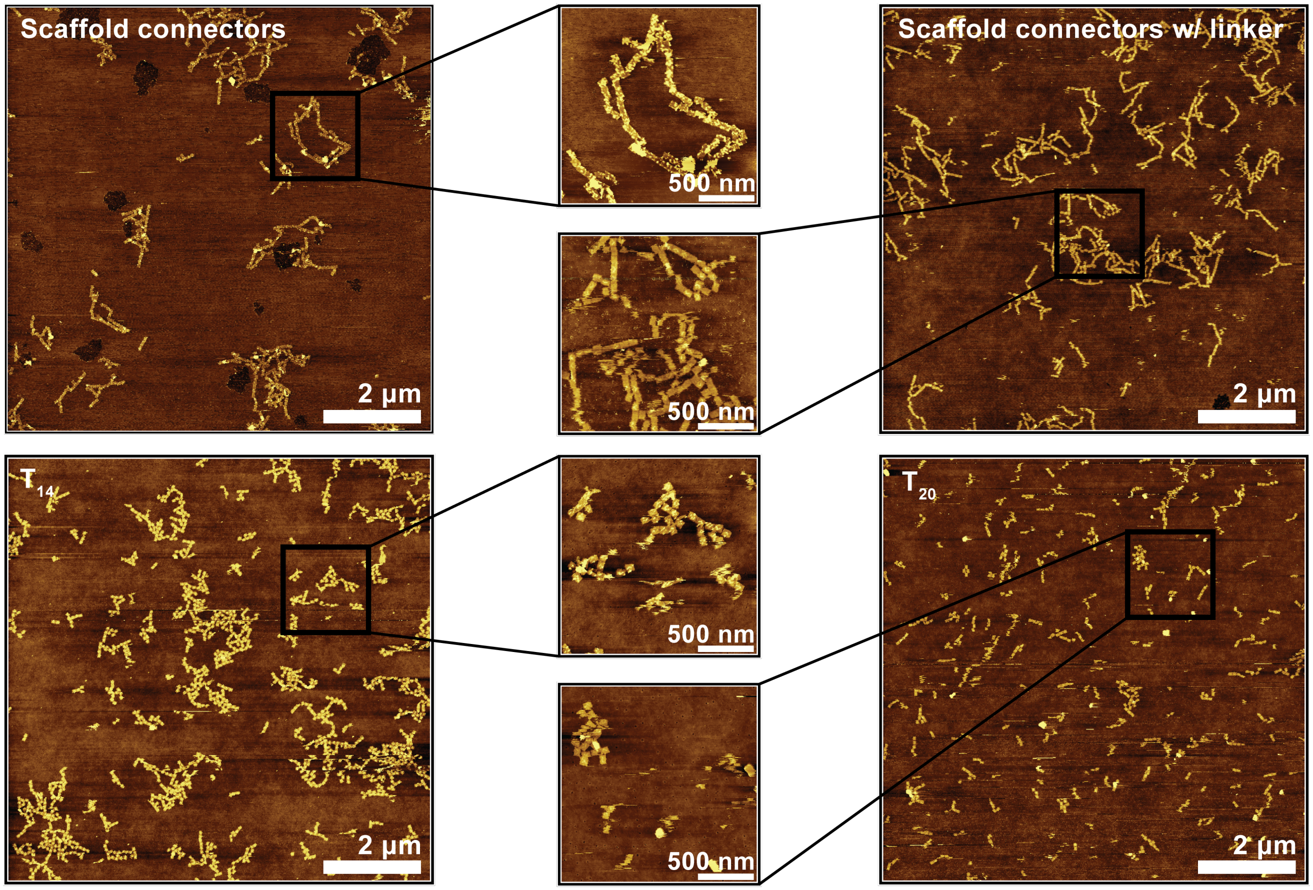

Figure S3: More AFM results. Shown are images comparable to those in main text Figure 3, but with different connector strands: Either scaffold connectors (20 h incubation, top) or short repeat connectors (2 h incubation, bottom). All images were acquired with 250 nM of the specified connector strand. Color-coded height scale in all panels in 6 nm.

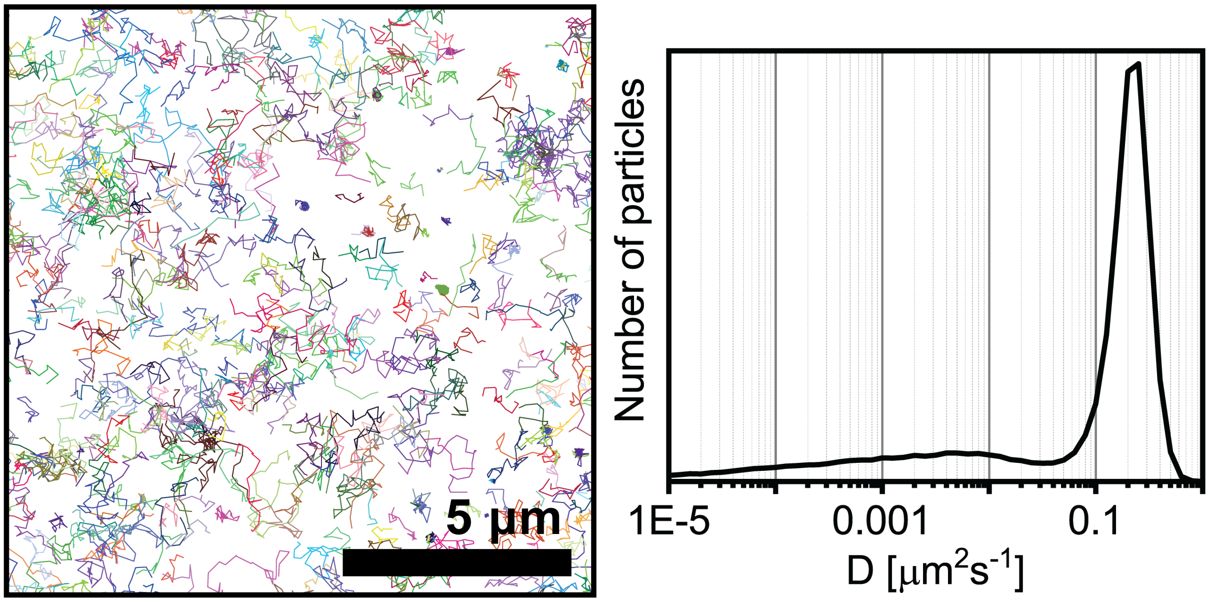

Figure S4: Example of single particle tracking results for monomeric DNA origami particles on DOPC SLBs. DNA origami particles are tethered to the membrane via 8 TEG-chol anchors. Track map (left) shows a random subset of tracks for better visibility. Colors are randomly assigned to individual tracks as a guide to the eye. The diffusion coefficient histogram to the right shows a minor peak at low mobility (trapped particles, a few of which are visible in the track map) and a pronounced peak of freely diffusing particles.

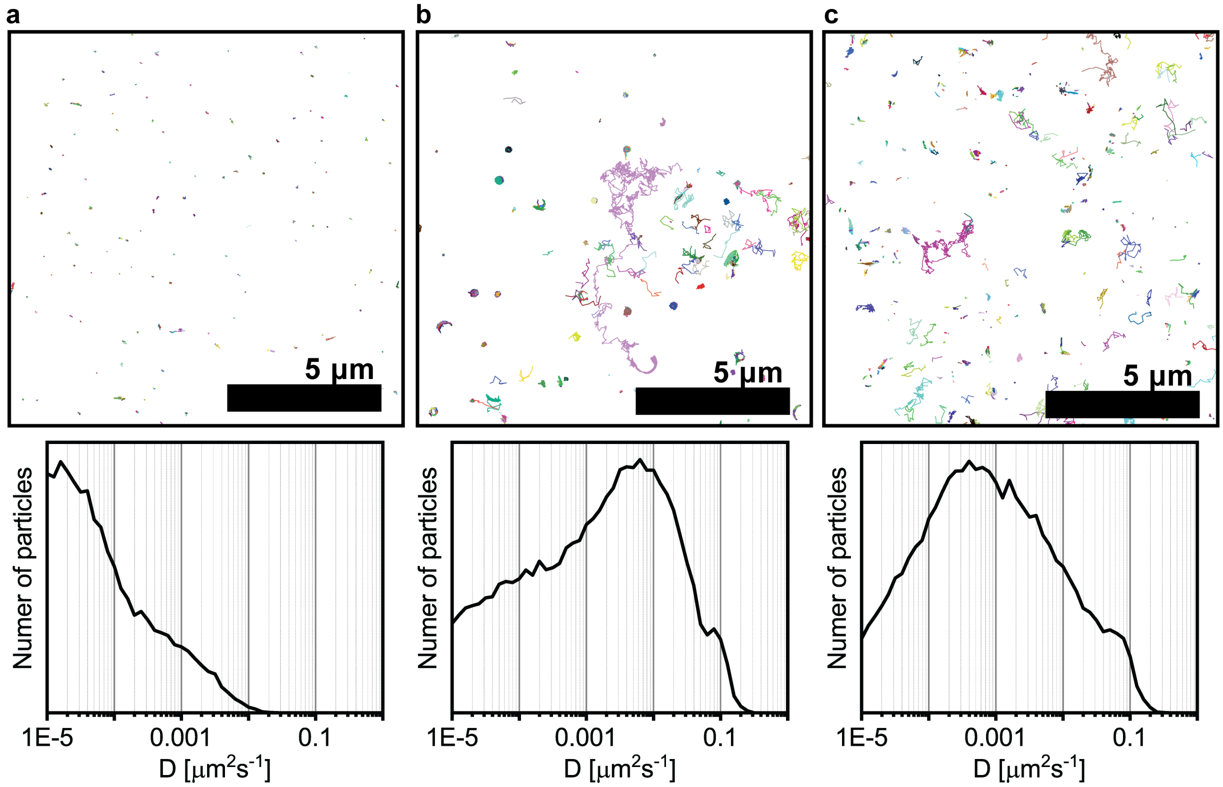

Figure S5: More single particle tracking results. Track maps are shown the same way as in Figure S4. **a:** Surface-immobilized (via streptavidin anchoring), tracking handle-labelled particles to assess the lower end of diffusion coefficients accessible via our method. **b:** Tracking handle-labelled DNA origami mixed and cross-linked (30 min, 50 nM each T_{14}, T_{20}, T_{40}, T_{60}, and T_{80}) with an excess of unlabelled particles (otherwise as in Figure S4). **c:** Same as panel b, but using scaffold connectors (with linker sequence, 20 h incubation before acquisition).

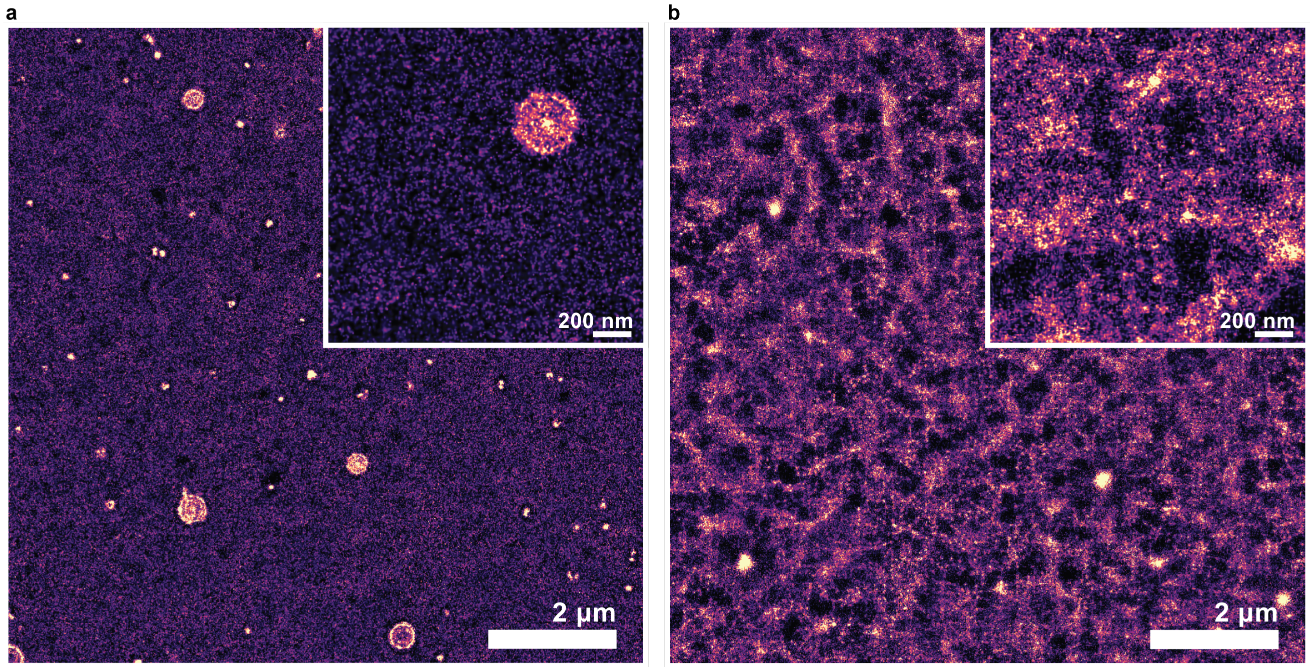

Figure S6: DNA-PAINT imaging of non-cross-linked DNA origami nanostructures. **a:** Similar conditions as in Figure 4, except without addition of connector strands. DNA-PAINT imaging shows a diffuse background from mobile particles, and a few immobilized particles at membrane defects (circular structures are larger holes in the supported lipid bilayer). **b:** Same as a, but with 3-fold higher density of DNA origami particles. Under such crowded conditions, particles are hindered in their motion and DNA-PAINT imaging reveals some structure, but with very low resolution.

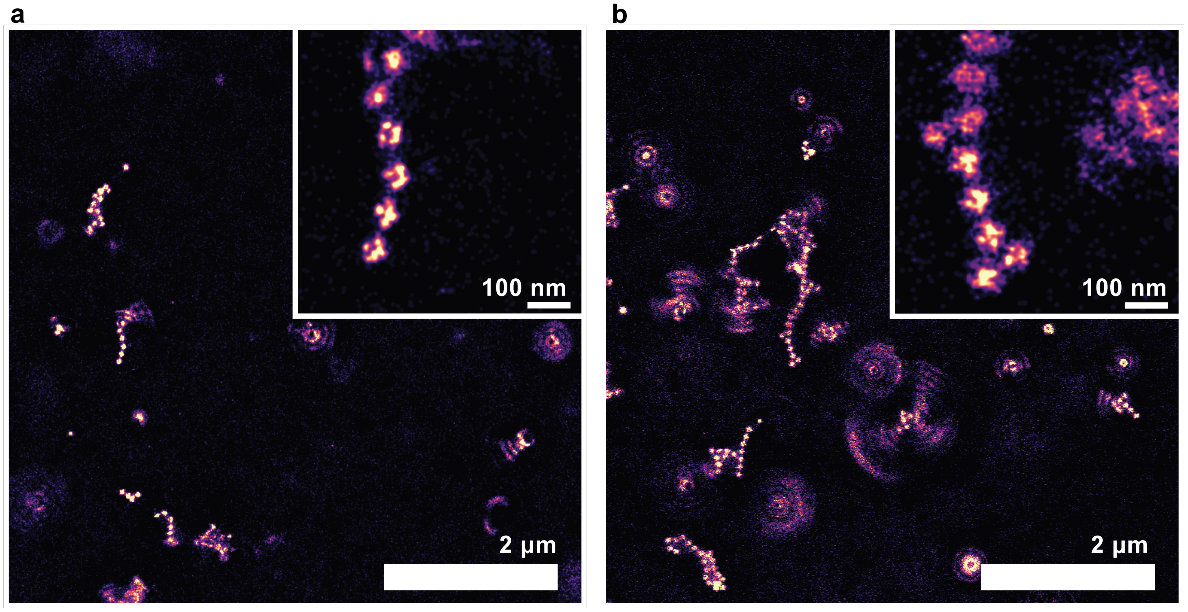

Figure S7: DNA-PAINT imaging of incompletely cross-linked DNA origami superstructures. Images have been acquired after 2 h cross-linking incubation with scaffold connectors without (**a**) or with (**b**) flexible linker sequence.

Supporting Tables

Table S1: Image analysis parameters.

Figure	Processing
1c overview	Localize MNG 450 box size 5; Render RCC undrift 450 frames, manual particle picking, pick similar, undrift from picked, export zoom 50 max density 1.7
1c average	Localize MNG 450 box size 5; Render RCC undrift 450 frames, manual particle picking, pick similar, undrift from picked, manual particle picking; Average3 Center of mass, oversampling 65, rotate, center of mass, rotate; Render export zoom 1000 max density 230
5,S1	tempVarianceAnalysis_v4.py with one frame lag, threshold_multiplication 0.5, bin_frames 3
4a overview	Localize MNG 500 box size 5; Render RCC undrift 450 frames, export zoom 7.5 max density 25
4a zoom	Localize MNG 500 box size 5; Render RCC undrift 450 frames, export zoom 100 max density 0.2
4b overview	Localize MNG 500 box size 5; Render RCC undrift 450 frames, export zoom 7.5 max density 30
4b zoom	Localize MNG 500 box size 5; Render RCC undrift 450 frames, export zoom 250 max density 0.15
4c T_{20} overview	Localize MNG 450 box size 5; Render RCC undrift 450 frames, export zoom 15 max density 8.33
4c T_{20} zoom	Localize MNG 450 box size 5; Render RCC undrift 450 frames, export zoom 250 max density 0.04

Continued on next page

Table S1

Figure	Processing
4c T_{40} overview	Localize MNG 450 box size 5; Render RCC undrift 450 frames, export zoom 15 max density 8
4c T_{40} zoom	Localize MNG 450 box size 5; Render RCC undrift 450 frames, export zoom 150 max density 0.12
4c T_{60} overview	Localize MNG 450 box size 5; Render RCC undrift 450 frames, export zoom 15 max density 8
4c T_{60} zoom	Localize MNG 450 box size 5; Render RCC undrift 450 frames, export zoom 150 max density 0.12
4c T_{80} overview	Localize MNG 450 box size 5; Render RCC undrift 450 frames, export zoom 15 max density 7.5
4c T_{80} zoom	Localize MNG 450 box size 5; Render RCC undrift 450 frames, export zoom 250 max density 0.035
S2	tempVarianceAnalysis_v4.py with one frame lag, threshold_multiplication 0.5, bin_frames 1 or 3
S4	Localize MNG 2500 box size 5; SPT.link_locs memory 2 search_range 3
S5a	Localize MNG 2500 box size 5; SPT.link_locs memory 2 search_range 1
S5b	Localize MNG 2500 box size 5; SPT.link_locs memory 2 search_range 2
S5c	Localize MNG 2500 box size 5; SPT.link_locs memory 2 search_range 2
S6a overview	Localize MNG 450 box size 5; Render RCC undrift 450 frames, export zoom 10 max density 5

Continued on next page

Table S1

Figure	Processing
S6a zoom	Localize MNG 450 box size 5; Render RCC undrift 450 frames, export zoom 60 max density 0.6
S6b overview	Localize MNG 450 box size 5; Render RCC undrift 450 frames, export zoom 10 max density 10
S6b zoom	Localize MNG 450 box size 5; Render RCC undrift 450 frames, export zoom 60 max density 0.25
S7a overview	Localize MNG 450 box size 5; Render RCC undrift 450 frames, export zoom 15 max density 7
S7a zoom	Localize MNG 450 box size 5; Render RCC undrift 450 frames, export zoom 150 max density 0.15
S7b overview	Localize MNG 450 box size 5; Render RCC undrift 450 frames, export zoom 15 max density 6
S7b zoom	Localize MNG 450 box size 5; Render RCC undrift 450 frames, export zoom 150 max density 0.08

Table S2: Unfunctionalized staple strands used in all experiments.

Name	Sequence
0[47]1[31]	AGAAAGGAACAACTAAAGGAATTCAAAAAAA
0[79]1[63]	ACAACTTTCAACAGTTTCAGCGGATGTATCGG
0[111]1[95]	TAAATGAATTTTCTGTATGGGATTAATTTCTT
0[143]1[127]	TCTAAAGTTTTGTCGTCTTTCCAGCCGACAA
0[175]0[144]	TCCACAGACAGCCCTCATAGTTAGCGTAACGA
0[207]1[191]	TCACCAGTACAAACTACAACGCCTAGTACCAG
0[239]1[223]	AGGAACCCATGTACCGTAACACTTGATATAA
0[271]1[255]	CCACCCTCATTTTCAGGGATAGCAACCGTACT
1[32]3[31]	AGGCTCCAGAGGCTTTGAGGACACGGGTAA
1[64]4[64]	TTTATCAGGACAGCATCGGAACGACACCAACC-TAAAACGAGGTCAATC
1[96]3[95]	AAACAGCTTTTTGCGGGATCGTCAACACTAAA
1[128]4[128]	TGACAACTCGCTGAGGCTTGCATTATAC-CAAGCGCGATGATAAA
1[160]2[144]	TTAGGATTGGCTGAGACTCCTCAATAACCGAT
1[192]4[192]	GCGGATAACCTATTATTCTGAAACAGACGATTG-GCCTTGAAGAGCCAC
1[224]3[223]	GTATAGCAAACAGTTAATGCCCAATCCTCA
1[256]4[256]	CAGGAGGTGGGGTCAGTGCCTTGAGTCTCT-GAATTTACCGGGAACCAG
2[47]0[48]	ACGGCTACAAAAGGAGCCTTTAATGTGAGAAT
2[79]0[80]	CAGCGAAACTTGCTTTCGAGGTGTTGCTAA
2[111]0[112]	AAGGCCGCTGATACCGATAGTTGCGACGTTAG
2[143]1[159]	ATATTCGGAACCATCGCCCACGCAGAGAAGGA

Continued on next page

Table S2

**Name**	**Sequence**
2[175]0[176]	TATTAAGAAGCGGGGTTTTGCTCGTAGCAT
2[207]0[208]	TTTCGGAAGTGCCGTCGAGAGGGTGAGTTTCG
2[239]0[240]	GCCCGTATCCGGAATAGGTGTATCAGCCCAAT
2[271]0[272]	GTTTTAACTTAGTACCGCCACCCAGAGCCA
3[32]5[31]	AATACGTTTGAAAGAGGACAGACTGACCTT
3[96]5[95]	ACACTCATCCATGTTACTTAGCCGAAAGCTGC
3[160]4[144]	TTGACAGGCCACCACCAGAGCCGCGATTTGTA
3[224]5[223]	TTAAAGCCAGAGCCGCCACCCTCGACAGAA
4[47]2[48]	GACCAACTAATGCCACTACGAAGGGGGTAGCA
4[79]2[80]	GCGCAGACAAGAGGCAAAAGAATCCCTCAG
4[111]2[112]	GACCTGCTCTTTGACCCCCAGCGAGGGAGTTA
4[143]3[159]	TCATCGCCAACAAAGTACAACGGACGCCAGCA
4[175]2[176]	CACCAGAAAGGTTGAGGCAGGTCATGAAAG
4[207]2[208]	CCACCCTCTATTCACAAACAAATACCTGCCTA
4[239]2[240]	GCCTCCCTCAGAATGGAAAGCGCAGTAACAGT
4[271]2[272]	AAATCACCTTCCAGTAAGCGTCAGTAATAA
5[32]7[31]	CATCAAGTAAAACGAACTAACGAGTTGAGA
5[96]7[95]	TCATTCAGATGCGATTTTAAGAACAGGCATAG
5[224]7[223]	TCAAGTTTCATTAAAGGTGAATATAAAAGA
6[47]4[48]	TACGTTAAAGTAATCTTGACAAGAACCGAACT
6[79]4[80]	TTATACCACCAAATCAACGTAACGAACGAG
6[111]4[112]	ATTACCTTTGAATAAGGCTTGCCCAAATCCGC
6[175]4[176]	CAGCAAAAGGAAACGTCACCAATGAGCCGC
6[207]4[208]	TCACCGACGCACCGTAATCAGTAGCAGAACCG

Continued on next page

Table S2

Name	Sequence
6[239]4[240]	GAAATTATTGCCTTTAGCGTCAGACCGGAACC
6[271]4[272]	ACCGATTGTCGGCATTTTCGGTCATAATCA
7[32]9[31]	TTTAGGACAAATGCTTTAAACAATCAGGTC
7[56]9[63]	ATGCAGATACATAACGGGAATCGT-<br>CATAAATAAAGCAAAG
7[96]9[95]	TAAGAGCAAATGTTTAGACTGGATAGGAAGCC
7[224]9[223]	AACGCAAAGATAGCCGAACAAACCCTGAAC
7[248]9[255]	GTTTATTTTGTCACAATCTTACCGAAGCCCTT-<br>TAATATCA
8[47]6[48]	ATCCCCCTATACCACATTCAACTAGAAAAATC
8[79]6[80]	AATACTGCCCAAAAGGAATTACGTGGCTCA
8[111]6[112]	AATAGTAAACACTATCATAACCCTCATTGTGA
8[207]6[208]	AAGGAAACATAAAGGTGGCAACATTATCACCG
8[239]6[240]	AAGTAAGCAGACACCACGGAATAATATTGACG
8[271]6[272]	AATAGCTATCAATAGAAAATTCAACATTCA
9[32]11[31]	TTTACCCCAACATGTTTTAAATTTCCATAT
9[64]11[63]	CGGATTGCAGAGCTTAATTGCTGAAACGAGTA
9[256]11[255]	GAGAGATAGAGCGTCTTTCCAGAGGTTTTGAA
10[47]8[48]	CTGTAGCTTGACTATTATAGTCAGTTCATTGA
10[79]8[80]	GATGGCTTATCAAAAAGATTAAGAGCGTCC
10[111]8[112]	TTGCTCCTTTCAAATATCGCGTTTGAGGGGGT
10[207]8[208]	ATCCCAATGAGAATTAACTGAACAGTTACCAG
10[239]8[240]	GCCAGTTAGAGGGTAATTGAGCGCTTTAAGAA
10[271]8[272]	ACGCTAACACCCACAAGAATTGAAAATAGC

Continued on next page

Table S2

Name	Sequence
11[32]13[31]	AACAGTTTTGTACCAAAAACATTTTATTTC
11[64]13[63]	GATTTAGTCAATAAAGCCTCAGAGAACCCTCA
11[128]13[127]	TTTGGGGATAGTAGTAGCATTAAAAGGCCG
11[160]12[144]	CCAATAGCTCATCGTAGGAATCATGGCATCAA
11[192]13[191]	TATCCGGTCTCATCGAGAACAAGCGACAAAAG
11[256]13[255]	GCCTTAAACCAATCAATAATCGGCACGCGCCT
12[47]10[48]	TAAATCGGGATTCCCAATTCTGCGATATAATG
12[79]10[80]	AAATTAAGTTGACCATTAGATACTTTTGCG
12[143]11[159]	TTCTACTACGCGAGCTGAAAAGGTTACCGCGC
12[239]10[240]	CTTATCATTCCCGACTTGCGGGAGCCTAATTT
12[271]10[272]	TGTAGAAATCAAGATTAGTTGCTCTTACCA
13[32]15[31]	AACGCAAAATCGATGAACGGTACCGGTTGA
13[64]15[63]	TATATTTTGTCATTGCCTGAGAGTGGAAGATT
13[128]15[127]	GAGACAGCTAGCTGATAAATTAATTTTTGT
13[160]14[144]	GTAATAAGTTAGGCAGAGGCATTTATGATATT
13[192]15[191]	GTAAAGTAATCGCCATATTTAACAAAACTTTT
13[256]15[255]	GTTTATCAATATGCGTTATACAAACCGACCGT
14[47]12[48]	AACAAGAGGGATAAAAATTTTTAGCATAAAGC
14[143]13[159]	CAACCGTTTCAAATCACCATCAATTCGAGCCA
14[175]12[176]	CATGTAATAGAATATAAAGTACCAAGCCGT
14[271]12[272]	TTAGTATCACAATAGATAAGTCCACGAGCA
15[32]17[31]	TAATCAGCGGATTGACCGTAATCGTAACCG
15[64]18[64]	GTATAAGCCAACCCGTCGGATTCTGAC-<br>GACAGTATCGGCCGCAAGGCG

Continued on next page

Table S2

Name	Sequence
15[96]17[95]	ATATTTTGGCTTTCATCAACATTATCCAGCCA
15[128]18[128]	TAAATCAAAATAATTCGCGTCTCG-
	GAAACCAGGCAAAGGGAAGG
15[160]16[144]	ATCGCAAGTATGTAAATGCTGATGATAGGAAC
15[192]18[192]	TCAAATATAACCTCCGGCTTAGGTAA-
	CAATTTCATTTGAAGGCGAATT
15[224]17[223]	CCTAAATCAAAATCATAGGTCTAAACAGTA
15[256]18[256]	GTGATAAAAAGACGCTGAGAAGAGATAAC-
	CTTGCTTCTGTTCGGGAGA
16[47]14[48]	ACAAACGGAAAAGCCCCAAAAACACTGGAGCA
16[143]15[159]	GCCATCAAGCTCATTTTTTAACCACAAATCCA
16[175]14[176]	TATAACTAACAAAGAACGCGAGAACGCCAA
16[271]14[272]	CTTAGATTTAAGGCGTTAAATAAAGCCTGT
17[32]19[31]	TGCATCTTTCCCAGTCACGACGGCCTGCAG
17[96]19[95]	GCTTTCCGATTACGCCAGCTGGCGGCTGTTTC
17[160]18[144]	AGAAAACAAAGAAGATGATGAAACAGGCTGCG
17[224]19[223]	CATAAATCTTTGAATACCAAGTGTTAGAAC
18[47]16[48]	CCAGGGTTGCCAGTTTGAGGGGACCCGTGGGA
18[79]16[80]	GATGTGCTTCAGGAAGATCGCACAATGTGA
18[111]16[112]	TCTTCGCTGCACCGCTTCTGGTGCGGCCTTCC
18[143]17[159]	CAACTGTTGCGCCATTCGCCATTCAAACATCA
18[175]16[176]	CTGAGCAAAAATTAATTACATTTTGGGTTA
18[207]16[208]	CGCGCAGATTACCTTTTTTAATGGGAGAGACT
18[239]16[240]	CCTGATTGCAATATATGTGAGTGATCAATAGT

Continued on next page

Table S2

Name	Sequence
18[271]16[272]	CTTTTACAAAATCGTCGCTATTAGCGATAG
19[32]21[31]	GTCGACTTCGGCCAACGCGCGGGGTTTTTC
19[96]21[95]	CTGTGTGATTGCGTTGCGCTCACTAGAGTTGC
19[224]21[223]	CTACCATAGTTTGAGTAACATTTAAAATAT
20[47]18[48]	TTAATGAACTAGAGGATCCCCGGGGGGTAACG
20[79]18[80]	TTCCAGTCGTAATCATGGTCATAAAAGGGG
20[111]18[112]	CACATTAAAATTGTTATCCGCTCATGCGGGCC
20[175]18[176]	ATTATCATTCAATATAATCCTGACAATTAC
20[207]18[208]	GCGGAACATCTGAATAATGGAAGGTACAAAAT
20[239]18[240]	ATTTTAAAATCAAAATTATTTGCACGGATTCG
20[271]18[272]	CTCGTATTAGAAATTGCGTAGATACAGTAC
21[32]23[31]	TTTTCACTCAAAGGGCGAAAAACCATCACC
21[56]23[63]	AGCTGATTGCCCTTCAGAGTCCACTAT-TAAAGGGTGCCGT
21[96]23[95]	AGCAAGCGTAGGGTTGAGTGTTGTAGGGAGCC
21[120]23[127]	CCCAGCAGGCGAAAAATCCCTTATAAAT-CAAGCCGGCG
21[184]23[191]	TCAACAGTTGAAAGGAGCAAATGAAAAATCTA-GAGATAGA
21[224]23[223]	CTTTAGGGCCTGCAACAGTGCCAATACGTG
21[248]23[255]	AGATTAGAGCCGTCAAAAAACAGAGGTGAGGC-CTATTAGT
22[47]20[48]	CTCCAACGCAGTGAGACGGGCAACCAGCTGCA
22[79]20[80]	TGGAACAACCGCCTGGCCCTGAGGCCCGCT

Continued on next page

Table S2

**Name**	**Sequence**
22[111]20[112]	GCCCGAGAGTCCACGCTGGTTTGCAGCTAACT
22[207]20[208]	AGCCAGCAATTGAGGAAGGTTATCATCATTTT
22[239]20[240]	TTAACACCAGCACTAACAACTAATCGTTATTA
22[271]20[272]	CAGAAGATTAGATAATACATTTGTCGACAA
23[32]22[48]	CAAATCAAGTTTTTTGGGGTCGAAACGTGGA
23[64]22[80]	AAAGCACTAAATCGGAACCCTAATCCAGTT
23[96]22[112]	CCCGATTTAGAGCTTGACGGGGAAAAAGAATA
23[192]22[208]	ACCCTTCTGACCTGAAAGCGTAAGACGCTGAG
23[224]22[240]	GCACAGACAATATTTTTGAATGGGGTCAGTA
23[256]22[272]	CTTTAATGCGCGAACTGATAGCCCCACCAG

Table S3: Docking sites for DNA-PAINT.

Name	Sequence
5[160]6[144] PAINT	GCAAGGCCTCACCAGTAGCACCATGGGCTTGA TT TCCTCCTCCTCCTCCTCCT
6[143]5[159] PAINT	GATGGTTTGAACGAGTAGTAAATTTACCATTA TT TCCTCCTCCTCCTCCTCCT
7[120]9[127] PAINT	CGTTTACCAGACGACAAAGAAGTTTTGC-CATAATTCGA TT TCCTCCTCCTCCTCCTCCT
7[160]8[144] PAINT	TTATTACGAAGAACTGGCATGATTGCGAGAGG TT TCCTCCTCCTCCTCCTCCT
7[184]9[191] PAINT	CGTAGAAAATACATACCGAGGAAACGCAATAA-GAAGCGCA TT TCCTCCTCCTCCTCCTCCT
8[143]7[159] PAINT	CTTTTGCAGATAAAAACCAAAATAAAGACTCC TT TCCTCCTCCTCCTCCTCCT
8[175]6[176] PAINT	ATACCCAACAGTATGTTAGCAAATTAGAGC TT TCCTCCTCCTCCTCCTCCT
9[96]11[95] PAINT	CGAAAGACTTTGATAAGAGGTCATATTTCGCA TT TCCTCCTCCTCCTCCTCCT
9[128]11[127] PAINT	GCTTCAATCAGGATTAGAGAGTTATTTTCA TT TCCTCCTCCTCCTCCTCCT
9[160]10[144] PAINT	AGAGAGAAAAAAATGAAAATAGCAAGCAAACT TT TCCTCCTCCTCCTCCTCCT
9[192]11[191] PAINT	TTAGACGGCCAAATAAGAAACGATAGAAGGCT TT TCCTCCTCCTCCTCCTCCT
9[224]11[223] PAINT	AAAGTCACAAAATAAACAGCCAGCGTTTTA TT TCCTCCTCCTCCTCCTCCT

Continued on next page

Table S3

Name	Sequence
10[143]9[159] PAINT	CCAACAGGAGCGAACCAGACCGGAGCCTTTAC TT TCCTCCTCCTCCTCCTCCT
10[175]8[176] PAINT	TTAACGTCTAACATAAAAACAGGTAACGGA TT TCCTCCTCCTCCTCCTCCT
11[96]13[95] PAINT	AATGGTCAACAGGCAAGGCAAAGAGTAATGTG TT TCCTCCTCCTCCTCCTCCT
11[224]13[223] PAINT	GCGAACCTCCAAGAACGGGTATGACAATAA TT TCCTCCTCCTCCTCCTCCT
12[111]10[112] PAINT	TAAATCATATAACCTGTTTAGCTAACCTTTAA TT TCCTCCTCCTCCTCCTCCT
12[207]10[208] PAINT	GTACCGCAATTCTAAGAACGCGAGTATTATTT TT TCCTCCTCCTCCTCCTCCT
13[96]15[95] PAINT	TAGGTAAACTATTTTTGAGAGATCAAACGTTA TT TCCTCCTCCTCCTCCTCCT
13[224]15[223] PAINT	ACAACATGCCAACGCTCAACAGTCTTCTGA TT TCCTCCTCCTCCTCCTCCT
14[79]12[80] PAINT	GCTATCAGAAATGCAATGCCTGAATTAGCA TT TCCTCCTCCTCCTCCTCCT
14[111]12[112 PAINT]	GAGGGTAGGATTCAAAAGGGTGAGACATCCAA TT TCCTCCTCCTCCTCCTCCT
14[207]12[208 PAINT]	AATTGAGAATTCTGTCCAGACGACTAAACCAA TT TCCTCCTCCTCCTCCTCCT
14[239]12[240] PAINT	AGTATAAAGTTCAGCTAATGCAGATGTCTTTC TT TCCTCCTCCTCCTCCTCCT

Continued on next page

Table S3

**Name**	**Sequence**
16[79]14[80] PAINT	GCGAGTAAAAATATTTAAATTGTTACAAAG TT TCCTCCTCCTCCTCCTCCT
16[111]14[112] PAINT	TGTAGCCATTAAAATTCGCATTAAATGCCGGA TT TCCTCCTCCTCCTCCTCCT
16[207]14[208] PAINT	ACCTTTTTATTTTAGTTAATTTCATAGGGCTT TT TCCTCCTCCTCCTCCTCCT
16[239]14[240] PAINT	GAATTTATTTAATGGTTTGAAATATTCTTACC TT TCCTCCTCCTCCTCCTCCT
19[160]20[144] PAINT	GCAATTCACATATTCCTGATTATCAAAGTGTA TT TCCTCCTCCTCCTCCTCCT
20[143]19[159] PAINT	AAGCCTGGTACGAGCCGGAAGCATAGATGATG TT TCCTCCTCCTCCTCCTCCT
21[160]22[144] PAINT	TCAATATCGAACCTCAAATATCAATTCCGAAA TT TCCTCCTCCTCCTCCTCCT
22[143]21[159] PAINT	TCGGCAAATCCTGTTTGATGGTGGACCCTCAA TT TCCTCCTCCTCCTCCTCCT
22[175]20[176] PAINT	ACCTTGCTTGGTCAGTTGGCAAAGAGCGGA TT TCCTCCTCCTCCTCCTCCT
23[128]23[159] PAINT	AACGTGGCGAGAAAGGAAGGGAAACCAGTAA TT TCCTCCTCCTCCTCCTCCT
23[160]22[176] PAINT	TAAAAGGGACATTCTGGCCAACAAAGCATC TT TCCTCCTCCTCCTCCTCCT
12[175]10[176] PAINT	TTTTATTTAAGCAAATCAGATATTTTTTGT TT TCCTCCTCCTCCTCCTCCT

Table S4: Unfunctionalized staples for SPT (replacing unused DNA-PAINT docking sites).

Name	Sequence
5[160]6[144]	GCAAGGCCTCACCAGTAGCACCATGGGCTTGA
6[143]5[159]	GATGGTTTGAACGAGTAGTAAATTTACCATTA
7[120]9[127]	CGTTTACCAGACGACAAAGAAGTTTTGC-CATAATTCGA
7[160]8[144]	TTATTACGAAGAACTGGCATGATTGCGAGAGG
7[184]9[191]	CGTAGAAAATACATACCGAGGAAACGCAATAA-GAAGCGCA
8[143]7[159]	CTTTTGCAGATAAAAACCAAAATAAAGACTCC
8[175]6[176]	ATACCCAACAGTATGTTAGCAAATTAGAGC
9[96]11[95]	CGAAAGACTTTGATAAGAGGTCATATTTCGCA
9[128]11[127]	GCTTCAATCAGGATTAGAGAGTTATTTTCA
9[160]10[144]	AGAGAGAAAAAAATGAAAATAGCAAGCAAACT
9[192]11[191]	TTAGACGGCCAAATAAGAAACGATAGAAGGCT
9[224]11[223]	AAAGTCACAAAATAAACAGCCAGCGTTTTA
10[143]9[159]	CCAACAGGAGCGAACCAGACCGGAGCCTTTAC
10[175]8[176]	TTAACGTCTAACATAAAAACAGGTAACGGA
11[96]13[95]	AATGGTCAACAGGCAAGGCAAAGAGTAATGTG
11[224]13[223]	GCGAACCTCCAAGAACGGGTATGACAATAA
12[111]10[112]	TAAATCATATAACCTGTTTAGCTAACCTTTAA
12[207]10[208]	GTACCGCAATTCTAAGAACGCGAGTATTATTT
13[96]15[95]	TAGGTAAACTATTTTTGAGAGATCAAACGTTA
13[224]15[223]	ACAACATGCCAACGCTCAACAGTCTTCTGA
14[79]12[80]	GCTATCAGAAATGCAATGCCTGAATTAGCA

Continued on next page

Table S4

**Name**	**Sequence**
14[111]12[112]	GAGGGTAGGATTCAAAAGGGTGAGACATCCAA
14[207]12[208]	AATTGAGAATTCTGTCCAGACGACTAAACCAA
14[239]12[240]	AGTATAAAGTTCAGCTAATGCAGATGTCTTTC
16[79]14[80]	GCGAGTAAAAATATTTAAATTGTTACAAAG
16[111]14[112]	TGTAGCCATTAAAATTCGCATTAAATGCCGGA
16[207]14[208]	ACCTTTTTATTTTAGTTAATTTCATAGGGCTT
16[239]14[240]	GAATTTATTTAATGGTTTGAAATATTCTTACC
19[160]20[144]	GCAATTCACATATTCCTGATTATCAAAGTGTA
20[143]19[159]	AAGCCTGGTACGAGCCGGAAGCATAGATGATG
21[160]22[144]	TCAATATCGAACCTCAAATATCAATTCCGAAA
22[143]21[159]	TCGGCAAATCCTGTTTGATGGTGGACCCTCAA
22[175]20[176]	ACCTTGCTTGGTCAGTTGGCAAAGAGCGGA
23[128]23[159]	AACGTGGCGAGAAAGGAAGGGAAACCAGTAA
23[160]22[176]	TAAAAGGGACATTCTGGCCAACAAAGCATC

Table S5: Biotinylated staples for immobilization on glass.

Name	Sequence
4[63]6[56] Bio	[Bio]ATAAGGGAACCGGATATTCATTACGTCA-GGACGTTGGGAA
4[127]6[120] Bio	[Bio]TTGTGTCGTGACGAGAAACACCAAATT-TCAACTTTAAT
4[191]6[184] Bio	[Bio]CACCCTCAGAAACCATCGATAGCATTGA-GCCATTTGGGAA
4[255]6[248] Bio	[Bio]AGCCACCACTGTAGCGCGTTTTCAAGGG-AGGGAAGGTAAA
18[63]20[56] Bio	[Bio]ATTAAGTTTACCGAGCTCGAATTCGGGA-AACCTGTCGTGC
18[127]20[120] Bio	[Bio]GCGATCGGCAATTCCACACAACAGGTGC-CTAATGAGTG
18[191]20[184] Bio	[Bio]ATTCATTTTTGTTTGGATTATACTAAGAA-ACCACCAGAAG
18[255]20[248] Bio	[Bio]AACAATAACGTAAAACAGAAATAAAAATC-CTTTGCCCGAA

Table S6: TEG-chol anchor binding staples for membrane tethering.

Name	Sequence
4[63]6[56] Chol	TATGAGAAGTTAGGAATGTTAATAAGGGAAC-CGGATATTCATTACGTCAGGACGTTGGGAA
4[127]6[120] Chol	TATGAGAAGTTAGGAATGTTATTGTGTCGT-GACGAGAAACACCAAATTTCAACTTTAAT
4[191]6[184] Chol	TATGAGAAGTTAGGAATGTTACACCCTCA-GAAACCATCGATAGCATTGAGCCATTTGGGAA
4[255]6[248] Chol	TATGAGAAGTTAGGAATGTTAAGCCACCACTG-TAGCGCGTTTTCAAGGGAGGGAAGGTAAA
18[63]20[56] Chol	TATGAGAAGTTAGGAATGTTAATTAAGTTTACC-GAGCTCGAATTCGGGAAACCTGTCGTGC
18[127]20[120] Chol	TATGAGAAGTTAGGAATGTTAGCGATCG-GCAATTCCACACAACAGGTGCCTAATGAGTG
18[191]20[184] Chol	TATGAGAAGTTAGGAATGT-TAATTCATTTTTGTTTGGATTATACTAA-GAAACCACCAGAAG
18[255]20[248] Chol	TATGAGAAGTTAGGAATGTTAAACAATAACG-TAAAACAGAAATAAAAATCCTTTGCCCGAA

Table S7: A_7 extension staples.

Name	Sequence
0[303]1[295]	AAAAAAACAGAACCGCCACCCTCTCAGAAC-CGCCACCCT
1[8]0[0]	TCACGTTGAAAATCTCGCGAATAATAATTTT-TAAAAAAA
6[303]7[295]	AAAAAAACAAAGACAAAAGGGCGTATGGTT-TACCAGCGC
7[8]6[0]	GGTAGAAAGATTCATCGAACAACATTATTA-CAAAAAAAA
16[303]17[295]	AAAAAAAAATCCTTGAAAACATAATTAATTTTC-CCTTAG
17[8]16[0]	GTGTAGATGGGCGCATGGGATAGGT-CACGTTGAAAAAAA
22[303]23[295]	AAAAAAAAAAAATACCGAACGAACTAAAA-CATCGCCATT
23[8]22[0]	TGGCCCACTACGTGAACCGTCTATCAGGGC-GAAAAAAAA

Table S8: Connector strands.

Name	Sequence
T_{14}	TTTTTTTTTTTTTT
T_{20}	TTTTTTTTTTTTTTTTTTTT
T_{40}	TTTTTTTTTTTTTTTTTTTTTTTTTTTTTTTTT-TTTTTTT
T_{60}	TTTTTTTTTTTTTTTTTTTTTTTTTTTTTTTTT-TTTTTTTTTTTTTTTTTTTTTTTTTTT
T_{80}	TTTTTTTTTTTTTTTTTTTTTTTTTTTTTTTTT-TTTTTTTTTTTTTTTTTTTTTTTTTTTTTTTTT-TTTTTTTTTTTTTT
20nt_T_6	TTTTTTCCCCCCTTTTTT
40nt_T_6	TTTTTTCCCCCCCCCCCCCCCCCCCCCCCCC-CTTTTTT
60nt_T_6	TTTTTTCCCCCCCCCCCCCCCCCCCCCCCCCCC-CCCCCCCCCCCCCCCCCCCTTTTTT
80nt_T_6	TTTTTTCCCCCCCCCCCCCCCCCCCCCCCC-CCCCCCCCCCCCCCCCCCCCCCCCCCCCCC-CCCCCCCCCCCCTTTTTT
20nt_T_7	TTTTTTTCCCCCCTTTTTTT
40nt_T_7	TTTTTTTCCCCCCCCCCCCCCCCCCCCCCCCC-CTTTTTTT
60nt_T_7	TTTTTTTCCCCCCCCCCCCCCCCCCCCCCCCCC-CCCCCCCCCCCCCCCCCCCCTTTTTTT

Continued on next page

Table S8

Name	Sequence
80nt_T_7	TTTTTTTCCCCCCCCCCCCCCCCCCCCCCC-<br>CCCCCCCCCCCCCCCCCCCCCCCCCCCCCC-<br>CCCCCCCCCCCCCTTTTTTT
20nt_T_8	TTTTTTTTCCCCCCTTTTTTTT
40nt_T_8	TTTTTTTTCCCCCCCCCCCCCCCCCCCCCCCCC-<br>CTTTTTTTT
60nt_T_8	TTTTTTTTCCCCCCCCCCCCCCCCCCCCCCCCC-<br>CCCCCCCCCCCCCCCCCCCCCTTTTTTTT
80nt_T_8	TTTTTTTTCCCCCCCCCCCCCCCCCCCCCC-<br>CCCCCCCCCCCCCCCCCCCCCCCCCCCCCC-<br>CCCCCCCCCCCCCCTTTTTTTT
20nt_T_9	TTTTTTTTTCCCCCCTTTTTTTTT
40nt_T_9	TTTTTTTTTCCCCCCCCCCCCCCCCCCCCCCCC-<br>CCTTTTTTTTT
60nt_T_9	TTTTTTTTTCCCCCCCCCCCCCCCCCCCCCCCC-<br>CCCCCCCCCCCCCCCCCCCCCCTTTTTTTTT
80nt_T_9	TTTTTTTTTCCCCCCCCCCCCCCCCCCCCC-<br>CCCCCCCCCCCCCCCCCCCCCCCCCCCCCC-<br>CCCCCCCCCCCCCCCTTTTTTTTT
ScaffoldIZ1L	GC GAA TAA TAA TTT TT CCCCCCC CAG AAC CGC CAC CCT C
ScaffoldIZ2L	GA ACA ACA TTA TTA CA AAAAAAA CAA AGA CAA AAG GGC C

Continued on next page

Table S8

Name	Sequence
ScaffoldIZ3L	GG GAT AGG TCA CGT TG AAAAAAA AAT CCT TGA AAA CAT A
ScaffoldIZ4L	CC GTC TAT CAG GGC GA AAAAAAA AAA AAT ACC GAA CGA ACT A
ScaffoldIZ1	GC GAA TAA TAA TTT TT CAG AAC CGC CAC CCT C
ScaffoldIZ2	GA ACA ACA TTA TTA CA CAA AGA CAA AAG GGC C
ScaffoldIZ3	GG GAT AGG TCA CGT TG AAT CCT TGA AAA CAT A
ScaffoldIZ4	CC GTC TAT CAG GGC GA AAA AAT ACC GAA CGA ACT A

Table S9: **Miscellaneous oligonucleotides.**

Name/Purpose	Sequence
TEG-Chol anchor	TAACATTCCTAACTTCTCATA[CHOL]
Docking site extension for SPT (12[175]10[176] A20)	TTTTATTTAAGCAAATCAGATATTTTTTGT TT AAGAAAGAAAAGAAGAAAAG
Tracking handle ([TCT]38-cA20)	TCT TCT TCT TCT TCT TCT TCT TCT TCT TCT TCT TCT TCT TCT TCT TCT TCT TCT TCT TCT TCT TCT TCT TCT TCT TCT TCT TCT TCT TCT TCT TCT TCT TCT TCT TCT TCT TCT CTT TTC TTC TTT TCT TTC TT
R1$_{6nt}$-Cy3B imager strand	GGAGGA-Cy3B
R1$_{18nt}$-Cy3B imager strand	GGAGGAGGAGGAGGAGGA-Cy3B
R5_s2$_{8nt}$-Cy3B imager strand	AGAAGAAG-Cy3B

References

(1) Kolin, D. L.; Wiseman, P. W. Advances in Image Correlation Spectroscopy: Measuring Number Densities, Aggregation States, and Dynamics of Fluorescently labeled Macromolecules in Cells. *Cell Biochemistry and Biophysics* **2007**, *49*, 141–164.

(2) Elson, E. L. In *Methods in enzymology*; Tetin, S. Y., Ed.; Academic Press, 2013; Vol. 518; pp 11–41.

(3) Widengren, J.; Mets, l. In *Single molecule detection in solution*; Zander, C., Enderlein, J.,

Keller, R. A., Eds.; Wiley-VCH Verlag GmbH & Co. KGaA: Weinheim, FRG, 2002; pp 69–120.

(4) Petrov, E. P.; Schwille, P. In *Standardization and quality assurance in fluorescence measurements II: Bioanalytical and biomedical applications*; Resch-Genger, U., Ed.; Springer Berlin Heidelberg: Berlin, Heidelberg, 2008; pp 145–197.

(5) Palmer, A.; Thompson, N. Molecular aggregation characterized by high order autocorrelation in fluorescence correlation spectroscopy. *Biophysical Journal* **1987**, *52*, 257–270.

(6) Sergeev, M.; Costantino, S.; Wiseman, P. W. Measurement of monomer-oligomer distributions via fluorescence moment image analysis. *Biophysical Journal* **2006**, *91*, 3884–3896.
